# The Prostaglandin EP4 Antagonist Vorbipiprant Combined with PD-1 Blockade for Refractory Microsatellite-Stable Metastatic Colorectal Cancer: A Phase Ib/IIa Trial

**DOI:** 10.1158/1078-0432.CCR-24-2611

**Published:** 2024-12-02

**Authors:** Filippo Pietrantonio, Federica Morano, Monica Niger, Filippo Ghelardi, Claudia Chiodoni, Michele Palazzo, Federico Nichetti, Paolo Manca, Eleonora Cristarella, Valentina Doldi, Nadia Zaffaroni, Giovanna Sabella, Nadia Brambilla, Elena Benincasa, Giampaolo Giacovelli, Cristina Vitalini, Federica Girolami, Lucio C. Rovati

**Affiliations:** 1 Department of Medical Oncology, Fondazione IRCCS Istituto Nazionale dei Tumori, Milan, Italy.; 2 Molecular Immunology Unit, Experimental Oncology Department, Fondazione IRCCS Istituto Nazionale dei Tumori, Milan, Italy.; 3 Computational Oncology, Molecular Diagnostics Program, National Center for Tumor Diseases (NCT) and German Cancer Research Center (DKFZ), Heidelberg, Germany.; 4 Department of Medicine, Memorial Sloan Kettering Cancer Center, New York, New York.; 5 Molecular Pharmacology Unit, Experimental Oncology Department, Fondazione IRCCS Istituto Nazionale dei Tumori, Milan, Italy.; 6 Department of Pathology, Fondazione IRCCS Istituto Nazionale dei Tumori, Milan, Italy.; 7 Department of Clinical Research, Rottapharm Biotech, Monza, Italy.; 8 School of Medicine, University of Milano–Bicocca, Milan, Italy.

## Abstract

**Purpose:**

Novel combinations are required to overcome resistance to immune checkpoint inhibitors in proficient mismatch repair (pMMR) or microsatellite-stable (MSS) metastatic colorectal cancer (mCRC). We aimed to determine whether vorbipiprant, a prostaglandin E2 receptor EP4 subtype antagonist, can convert immune-resistant mCRC into a tumor responsive to anti–PD-1 inhibition.

**Patients and Methods:**

This phase Ib/IIa prospective, open-label, single-arm trial followed a 3 + 3 dose-escalation and dose-optimization design. A total of 28 patients with chemorefractory pMMR/MSS mCRC were given dose-escalated oral vorbipiprant (30, 90, or 180 mg twice daily), along with biweekly intravenous balstilimab (3 mg/kg), an anti–PD-1 antibody. The primary endpoints included safety and the disease control rate (DCR). Secondary endpoints were the overall response rate, duration of response, progression-free survival, and overall survival.

**Results:**

No dose-limiting toxicities were observed. Of the 28 patients, seven (25%) experienced serious adverse events, but only one was attributed to vorbipiprant and one to balstilimab. The trial achieved a DCR of 50% observed across the entire cohort. In the subgroup of patients with liver metastases (*n* = 12), the DCR was 25%. The overall response rate was 11%, with three patients showing a partial response (median duration of response, 7.4 months). The median progression-free survival was 2.6 months, and the median overall survival was 14.2 months. Translational exploratory analyses suggested that vorbipiprant may boost response to anti–PD-1 in patients with immunogenic tumors.

**Conclusions:**

The combination of vorbipiprant and a PD-1 inhibitor (balstilimab) yielded sufficient activity in refractory pMMR/MSS mCRC, which is worthy of confirmation in future clinical trials in biomarker-enriched populations.

**Translational Relevance**

This work showed that the combination of the prostaglandin E2 receptor EP4 subtype inhibitor vorbipiprant plus an anti–PD-1 agent (balstilimab) achieved responses in patients with proficient mismatch repair/microsatellite-stable (MSS), low–tumor mutational burden, and chemorefractory metastatic colorectal cancer (mCRC). These results are important for future trials with this combination strategy in mCRC and other tumors, and expansion of this study at the recommended vorbipiprant dose is ongoing in other gastrointestinal cancers. Moreover, this study highlights that a one-size-fits-all strategy is not reasonable for proficient mismatch repair/MSS mCRC, and rational development of immunotherapy combinations is necessary. We conducted extensive translational analyses in tumor and plasma samples of enrolled patients and showed that immune-enriched MSS tumors are likely to respond to this combination. Therefore, patient selection will be key to advancing immunotherapy in MSS mCRC, and vorbipiprant plus anti–PD-1 therapy may be a well-tolerated immunotherapy backbone to be investigated in future trials in molecularly selected subgroups.

## Introduction

The prognosis of metastatic colorectal cancer (mCRC) remains unfavorable despite several recent advances (1), and there is urgent need for novel therapeutic strategies in the refractory setting (2, 3). Immune checkpoint inhibitors (ICI) are the standard of care in the subset of patients with mismatch repair–deficient or microsatellite instability–high mCRC (4). Unfortunately, most patients with mCRC have proficient mismatch repair (pMMR) or microsatellite-stable (MSS) tumors, with minimal benefit from first-generation ICI administered as single agents (5). Therefore, novel combination strategies are being explored to overcome the refractoriness to ICI in pMMR/MSS mCRC (5, 6).

Within these strategies, targeting the activity of immunosuppressive mediators like prostaglandin E2 (PGE2) in the tumor microenvironment is among the most promising (7, 8). The PGE2 receptor EP4 subtype (EP4) is strongly associated with tumor growth, invasion, and metastasis (9, 10). Consequently, EP4 receptor blockade has the potential to reactivate antitumor immunity, thereby enhancing the overall efficacy of cancer immunotherapy (11, 12).

Vorbipiprant (formerly CR6086) is a novel, potent, and selective EP4 receptor antagonist, serving as a targeted immunomodulator (13, 14). In preclinical studies, vorbipiprant successfully reverted the intrinsic resistance to ICI of a murine MSS colorectal cancer model (15). Based on these encouraging results, we designed a phase Ib/IIa clinical trial to evaluate the safety and efficacy of dose-escalated vorbipiprant in combination with the anti–PD-1 fully human IgG4 mAb balstilimab (16–18) in patients with refractory pMMR/MSS mCRC, also exploring potential predictors of response.

## Patients and Methods

### Study design and participants

This is a phase Ib/IIa prospective, open-label, single-arm trial. Enrollment started in December 2021 and concluded in January 2023. The cutoff date for this analysis was May 23, 2024 (i.e., at treatment discontinuation in all patients). The study was conducted at a single research hospital (Department of Medical Oncology, Fondazione IRCCS Istituto Nazionale dei Tumori, Milan, Italy). The trial followed a 3 + 3 dose-escalation design with cohort expansions for dose optimization. Patient eligibility criteria included ≥18 years of age, histologically confirmed pMMR/MSS mCRC, and refractoriness or intolerance to standard treatments including fluoropyrimidines, oxaliplatin, and irinotecan, as well as anti-EGFR or anti-VEGF therapies when clinically indicated. Additionally, candidates had an Eastern Cooperative Oncology Group performance status of 0 or 1 and sufficient organ function. The trial was approved by the relevant institutional review board and was conducted in accordance with the ethical guidelines of the Declaration of Helsinki and all applicable regulations. The trial was registered at ClinicalTrials.gov (NCT05205330). A Data and Safety Monitoring Committee periodically reviewed the safety and efficacy results. All participants provided written informed consent before enrollment. This study followed the reporting guidelines under the EQUATOR umbrella (19).

### Sample size and treatment protocol

The study aimed to include 27 patients, a sample size deemed adequate to meet the trial's primary endpoints, reflecting the empirical approach often used in dose-escalation and -optimization studies. A formal power calculation was not conducted, given the 3+3 dose-escalation design of the study and the difficulty to predict the size of each dose cohort that had to be based mainly on safety aspects. On the other hand, previous studies indicated an almost complete inefficacy of ICI monotherapy in this setting (4). Vorbipiprant was given orally in escalating twice-daily dosages of 30, 90, and 180 mg. Balstilimab 3 mg/kg was administered intravenously every 2 weeks. Combination treatment with vorbipiprant and balstilimab was scheduled to continue for up to 2 years or until disease progression, unacceptable toxicity, or death.

### Patient follow-up

Tumor assessment was performed using contrast-enhanced CT scans during the screening phase and subsequently at 8-week intervals for the first year and at 12-week intervals thereafter. Additional assessments were conducted if disease progression was clinically suspected. Tumor response and progression were determined using RECIST version 1.1 (20). Archival tumor tissue was mandatorily available at screening, and upon patient consent, further biopsies were planned 2 months after treatment start and at disease progression, if feasible. After treatment cessation, patients were asked to return for a clinic visit after 30 days. Thereafter, safety and survival were monitored quarterly through phone calls, emails, or clinic visits. Follow-up continued until patients' deaths, withdrawal of consent, loss to follow-up, or study termination, whichever occurred first.

### Primary endpoints

The primary endpoints were safety and the disease control rate (DCR) at 24 weeks, as per the RECIST v1.1 guidelines (20). Safety assessments involved identifying dose-limiting toxicities (DLT), monitoring adverse events (AE), laboratory examinations, vital signs and ECG, physical examinations, and Eastern Cooperative Oncology Group performance status. Treatment-emergent AE (TEAE) were graded according to the Common Terminology Criteria for Adverse Events version 5.0 (21). DLT were defined as grade ≥3 TEAE, including abnormal laboratory values considered by the investigator related to vorbipiprant, regardless of their relationship with balstilimab. They were collected during the first two treatment cycles (i.e., for 28 days), at each dose level. In the cohort expansions, AE were collected, but a DLT rule was not applied for dose escalation. Immune-related AE (irAE) were defined as treatment-related TEAE consistent with an immune-mediated mechanism and treated with corticosteroids, immunosuppressives, or hormone replacement.

The DCR was defined as the ratio of patients with stable disease (SD), partial response (PR), or complete response (CR) to the total number of patients treated. Despite the seminal study by Le and colleagues (4) indicating an overall response rate (ORR; i.e., the ratio of patients with PR and CR) of 0% with the anti–PD-1 pembrolizumab in pMMR/MSS mCRC, the DCR was 11% by the 20-week timepoint (4), thus highlighting the overall need of improving both activity endpoints in future proof-of-concept studies. Here, the primary activity endpoint was considered met if the DCR exceeded 15%, coupled with an acceptable safety profile, with at least one dose of vorbipiprant combined with balstilimab, but no power calculation was done considering the preliminary dose-finding nature of this study.

### Secondary endpoints

Secondary endpoints included the ORR, duration of response, progression-free survival (PFS), and overall survival (OS). The ORR was calculated as the proportion of patients who achieved PR or CR (20) of the total number of treated patients. The duration of response was measured as the time from the first documented instance of CR or PR until disease progression or death. PFS was determined as the time from the start of treatment to the first documentation of disease progression or death. OS was defined as the time from treatment start to death. For patients alive, OS data were censored at their last follow-up.

### Exploratory endpoints

Several exploratory biochemical and molecular assays were performed to unveil biological determinants of tumor immunogenicity and identify potential biomarkers of treatment efficacy. A detailed description of methods used for translational analyses is reported in the Supplementary Material as Supplementary Methods S1. Briefly, serial extensive immunophenotyping was performed on frozen peripheral blood mononuclear cells collected at baseline, 2-month (early), 6-month, and progressive disease timepoints. Cells were thawed and tested by multicolor flow-cytometry analysis to assess different immune cell populations. In addition, measurement of a panel of selected cytokines was performed on plasma samples collected at the same timepoints and analyzed using commercially available ELISA kits. RNA sequencing (RNA-seq) was performed on archival formalin-fixed, paraffin-embedded specimens from baseline biopsies or surgical samples of the primary tumor or metastases. To identify pathways enriched among genes correlated with treatment response, gene set enrichment analysis (GSEA; RRID: SCR_003199 + RRID: SCR_006442) was performed on the full list of differentially expressed genes, as evaluated via the *DESeq2* (RRID: SCR_015687 + RRID: SCR_006442) pipeline (22). Moreover, a curated list of immune-related inflammatory gene expression signatures was used as input to perform single-sample GSEA (RRID: SCR_021058 + RRID: SCR_006442) and assess the predictive role of each signature in treatment response (23, 24). Moreover, deconvolution algorithms were applied to investigate immune cell subpopulation enrichment in the tumor microenvironment. Count data were used to infer the gene expression–based consensus molecular subtypes (CMS; ref. 25) and the DetermaIO immune-related gene signature with the previously described cutoff for positivity (26). Finally, PD-L1 expression was assessed using the 22C3 pharmDx assay (RRID: AB_2889976) and measured using the combined positive score (CPS). Tumor mutational burden (TMB) was determined in patients with favorable objective response using the FoundationOne CDx test (RRID: SCR_025628).

### Statistical analysis

The median PFS and OS were computed with their 95% confidence intervals (CI) using the Kaplan–Meier method. The median follow-up (and IQR) was calculated as the time from treatment start to last contact or death. The Wilcoxon–Mann–Whitney and χ^2^ tests were adopted in the exploratory translational analyses to compare the distribution of continuous and categorical variables, respectively, between patients with PFS >4 months (i.e., confirmed PR or SD) and others, with *P* values adjusted for multiple comparisons through the Benjamini–Hochberg procedure, whereas the Wilcoxon signed-rank test was used to compare paired values. Logistic regression analysis was performed to identify significant predictors of treatment response, with statistical significance for these exploratory analyses set at a 10% two-sided threshold.

All statistical analyses were conducted using SAS version 9.4 (SAS Institute Inc.; RRID: SCR_008567) and R version 4.3.2 (Posit; RRID: SCR_001905).

### Data availability

Gene expression data have been deposited at the Gene Expression Omnibus (http://www.ncbi.nlm.nih.gov/geo; RRID: SCR_005012) with Gene Expression Omnibus accession number GSE281374.

Other data generated in this study are available from the corresponding author upon request.

## Results

### Patients and treatment allocation

**Figure 1.**
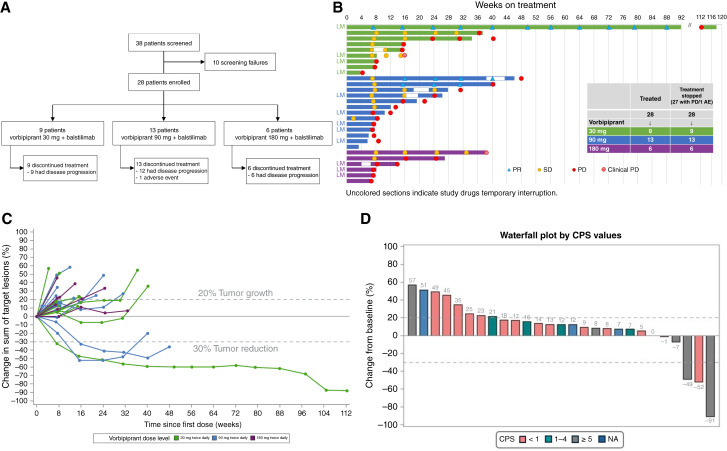
Trial profile and clinical response. Trial profile showing the number of patients screened, the patients enrolled, and, finally, the number of patients discontinued by the treatment groups (**A**). Swimmer plot showing the duration of treatment with vorbipiprant and balstilimab and RECIST overall tumor assessments (**B**). LM, liver metastases; PD, progressive disease. Spider plot showing the tumor burden over time presented as percentage change in the sum of the target lesions (**C**). Waterfall plot reporting treatment response according to PD-L1 CPS (**D**). NA, not assessable. Vorbipiprant dose always combined with the PD-1 inhibitor balstilimab, if not indicated.

During the 14-month recruitment period, 38 patients were screened and 28 were enrolled (Fig. 1A).

Patients’ characteristics are shown in Table 1 and the study representativeness in Supplementary Table S1.

**Table 1.** Baseline characteristics of the study patients.

Variable	Vorbipiprant 30 mg + balstilimab (*n* = 9)	Vorbipiprant 90 mg + balstilimab (*n* = 13)	Vorbipiprant 180 mg + balstilimab (*n* = 6)	Entire cohort (*n* = 28)
Age (years), median (range)	64 (40–70)	58 (41–76)	60 (49–69)	59 (40–76)
Sex, *n* (%)				
Male	3 (33%)	6 (46%)	6 (100%)	15 (54%)
Female	6 (67%)	7 (54%)	0	13 (46%)
Race, *n* (%)				
White	9 (100%)	13 (100%)	6 (100%)	28 (100%)
ECOG PS, *n* (%)				
0	7 (78%)	7 (54%)	6 (100%)	20 (71%)
1	2 (22%)	6 (46%)	0	8 (29%)
Primary tumor site, *n* (%)				
Right colon	4 (44%)	3 (23%)	0	7 (25%)
Left colon	2 (22%)	7 (54%)	2 (33%)	11 (39%)
Rectum	3 (33%)	3 (23%)	4 (67%)	10 (36%)
Prior lines of chemotherapy, median (range)	3 (2–7)	4 (2–10)	3.5 (3–8)	4 (2–10)
Liver metastases, *n* (%)				
Yes	4 (44%)	5 (38%)	3 (50%)	12 (43%)
No	5 (56%)	8 (62%)	3 (50%)	16 (57%)
*RAS* mutation status, *n* (%)				
Wild-type	3 (33%)	4 (31%)	3 (50%)	10 (36%)
Mutated	6 (67%)	9 (69%)	3 (50%)	18 (64%)
*BRAF* mutation status, *n* (%)				
Wild-type	8 (89%)	12 (92%)	6 (100%)	26 (93%)
Mutated	1 (11%)	1 (8%)	0	2 (7%)
Previous treatment, *n* (%)^a^				
Trifluridine/tipiracil	1 (11%)	3 (23%)	0	4 (14%)
Regorafenib	0	2 (15%)	1 (17%)	3 (11%)
Trifluridine/tipiracil and regorafenib	2 (22%)	4 (31%)	2 (33%)	8 (29%)

Vorbipiprant administered orally at the indicated dose given twice daily. Balstilimab administered intravenously at a dosage of 3 mg/kg every 2 weeks.

Abbreviation: ECOG PS, Eastern Cooperative Oncology Group performance status.

a All patients had previously been exposed to fluoropyrimidines, oxaliplatin, and irinotecan. Patients with wild-type *RAS* had also undergone prior EGFR treatment.

Active liver metastases were present in 12 (43%) patients at the time of enrollment, whereas nine patients had undergone prior resection of liver metastases. Patients had a median of four prior lines of chemotherapy (range, 2–10). There were no apparent intergroup differences in terms of baseline characteristics. Baseline safety data were normal or with nonclinically significant abnormalities. Vorbipiprant dose-escalation/expansion proceeded according to the protocol (Fig. 1A), with three patients initially enrolled in the 30-mg twice-daily group, 12 patients in the 90-mg twice-daily group plus one patient added because of an early discontinuation before the first efficacy assessment, and six patients in the 180-mg twice-daily group. In the context of dose optimization, this last cohort was not expanded to 12 patients as originally planned; however, six patients were added to the first 30-mg twice-daily combination group instead (via protocol amendment), based on the favorable safety and efficacy results observed in the meantime with this low-dose cohort.

As of the cutoff date, all 28 patients had discontinued treatment (Fig. 1A). Disease progression was the most common reason for discontinuation (*n* = 27). One patient in the 90-mg twice daily + balstilimab group discontinued treatment because of a serious AE not related to the study drugs.

### Primary endpoints

#### Safety

No DLT occurred during dose escalation.

A detailed summary of TEAE is provided in Table 2.

**Table 2.** Overview of TEAE.

Variable	Vorbipiprant 30 mg + balstilimab (*n* = 9)	Vorbipiprant 90 mg + balstilimab (*n* = 13)	Vorbipiprant 180 mg + balstilimab (*n* = 6)	Entire cohort (*n* = 28)
Number of DLT	0	0	0	0
Any TRAE	8 (89%)	9 (69%)	3 (50%)	20 (71%)
Grade ≥3 AE	2 (22%)	4 (31%)	1 (17%)	7 (25%)
Vorbipiprant-related grade ≥3 AE	1 (11%)	0	0	1 (4%)
Balstilimab-related grade ≥3 AE	0	1 (8%)	0	1 (4%)
Serious AE	3 (33%)	3 (23%)	1 (17%)	7 (25%)
Vorbipiprant-related serious AE	1 (11%)	0	0	1 (4%)
Balstilimab-related serious AE	1 (11%)	0	0	1 (4%)
AE leading to dropout	0	1 (8%)^a^	0	1 (4%)
Fatal AE	0	1 (8%)^a^	0	1 (4%)
TEAE with incidence >10% overall by MedDRA PT				
Blood alkaline phosphatase increased	2 (22%)	5 (38%)	4 (67%)	11 (39%)
γ-Glutamyltransferase increased	1 (11%)	3 (23%)	2 (33%)	6 (21%)
Cough	1 (11%)	5 (38%)	0	6 (21%)
Alanine aminotransferase increased	3 (33%)	1 (8%)	1 (17%)	5 (18%)
Aspartate aminotransferase increased	2 (22%)	1 (8%)	2 (33%)	5 (18%)
Fatigue	0	5 (38%)	0	5 (18%)
Anemia	1 (11%)	2 (15%)	1 (17%)	4 (14%)
Diarrhea	1 (11%)	2 (15%)	1 (17%)	4 (14%)
Hypothyroidism	1 (11%)	3 (23%)	0	4 (14%)
Urinary tract infection	2 (22%)	2 (15%)	0	4 (14%)
Abdominal pain	0	3 (23%)	0	3 (11%)
Abdominal pain upper	2 (22%)	1 (8%)	0	3 (11%)
Back pain	0	3 (23%)	0	3 (11%)
Blood creatine phosphokinase increased	2 (22%)	0	1 (17%)	3 (11%)
Dyspnea	0	2 (15%)	1 (17%)	3 (11%)
Vomiting	1 (11%)	2 (15%)	0	3 (11%)

Vorbipiprant administered orally at the indicated dose given twice daily. Balstilimab administered intravenously at a dosage of 3 mg/kg every 2 weeks.

Data are presented as frequencies with corresponding percentages in parentheses. AE were coded using MedDRA version 24.1.

Abbreviations: MedDRA PT, Medical Dictionary for Regulatory Activities Preferred Terms; TRAE, treatment-related adverse events.

a Pulmonary embolism due to disease progression.

Of the 28 patients, seven (25%) experienced serious TEAE, but only two cases were treatment related. Specifically, vorbipiprant was associated with a duodenal ulcer hemorrhage in one patient in the 30-mg twice-daily group, prompting the use of prophylactic proton pump inhibitors for all subsequent participants. Another patient developed an immune-related grade 2 pneumonitis related to balstilimab. There were no treatment-related deaths. One patient died because of a pulmonary embolism related to disease progression and was the sole participant to be withdrawn for AE. The most common TEAE was elevated blood alkaline phosphatase (39% of patients) of grade 2 maximum severity and with no signs or symptoms of cholestasis. There were no safety concerns from irAE, with medical treatment required only in six (21%) patients. No significant changes were noted in other laboratory tests, blood pressure, heart rate, physical exams, or ECG findings. Hence, vorbipiprant in combination with the PD-1 inhibitor balstilimab was well tolerated across doses.

#### Efficacy

An overview of best overall responses and DCR is presented in Table 3.

**Table 3.** Summary of best overall responses and DCR.

Best overall response	Vorbipiprant 30 mg + balstilimab (*n* = 9)	Vorbipiprant 90 mg + balstilimab (*n* = 13)	Vorbipiprant 180 mg + balstilimab (*n* = 6)	Entire cohort (*n* = 28)	Patients with liver metastases (*n* = 12)
CR	0	0	0	0	0
PR	1 (11%)	2 (15%)	0	3 (11%)	1 (8%)
SD	5 (56%)	4 (31%)	2 (33%)	11 (39%)	2 (17%)
PD	3 (33%)	6 (46%)	4 (67%)	13 (46%)	9 (75%)
Not evaluable	0	1 (8%)	0	1 (4%)	0
DCR (CR + PR + SD)	67%	46%	33%	50%	25%

Abbreviation: PD, progressive disease.

Data are presented as frequencies with corresponding percentages in parentheses. Vorbipiprant administered orally at the indicated dose given twice daily. Balstilimab administered intravenously at a dosage of 3 mg/kg every 2 weeks.

The trial met its designated primary endpoint, with a DCR exceeding 15% in the entire study cohort (DCR = 50%) and within each vorbipiprant dose combination group. The DCR were 25% in patients with liver metastases and 69% in patients without (*n* = 16).

### Secondary endpoints

The ORR was 11%, with three patients showing PR with this combined treatment (1/9 with vorbipiprant 30 mg twice daily and 2/13 with 90 mg twice daily; Table 3): All patients had TMB-low (<10 mutations/Mb) status and no pathogenic *POLE/D1* proofread domain mutations. The median duration of response was 7.4 months (95% CI, 5.7–24.2). Single patient data for treatment duration and response are reported in Fig. 1B (swimmer plot), tumor burden over time in Fig. 1C (spider plot), and change from baseline in Fig. 1D (waterfall plot).

The first patient’s PR persisted for 24.2 months; this patient had liver metastases upon enrollment. In the other two patients with PR, this lasted for 7.4 and 5.7 months, respectively. Among the 11 patients whose best response was SD, all experienced disease control for at least 12 weeks (median, 16.4 weeks; range, 13.6–38.7 weeks), with five maintaining it for 24 weeks or longer. Regarding survival outcomes, the median follow-up was 13.8 months (IQR, 7.7–18.3). The median PFS was 2.6 months (95% CI, 1.7–3.6), and the median OS was 14.2 months [95% CI, 10.6–not reached (NR); Supplementary Fig. S1A and S1B]. Patients without liver metastases had a median PFS of 3.6 months (95% CI, 1.8–7.2), and the median OS was NR (95% CI, 12.8–NR). In patients with liver metastases, the median PFS was 1.8 months (95% CI, 1.6–3.5), and the median OS was 13.7 months (95% CI, 5.5–15.1; Supplementary Fig. S1C and S1D). Most of the evaluable patients (19 of 27, i.e., 70%) received postprogression subsequent systemic anticancer therapy, including trifluridine/tipiracil, regorafenib, temozolomide, or re-treatment with panitumumab, irinotecan, capecitabine, and FOLFOX with or without bevacizumab.

### Exploratory endpoints

Immunophenotyping of peripheral blood mononuclear cells showed that patients with PFS >4 months (*n* = 8) had a significantly lower relative abundance of PD-1^+^ regulatory T cells and PD-1^+^ CD4 cells at baseline (Supplementary Fig. S2A) and significant early expansion of GRZMB^+^ Ki67^+^ CD8 by the 2-month timepoint (Supplementary Fig. S2B), without differences among doses (Supplementary Fig. S2C). A statistically significant increase in the T-cell–recruiting chemokine CXCL10 levels in plasma was observed after 2-month treatment in the entire population across doses (*P* < 0.0001; Supplementary Fig. S2D), correlating with an increase in CD8^+^CD3^+^ cells (Spearman value 0.39; *P* < 0.05).

**Figure 2.**
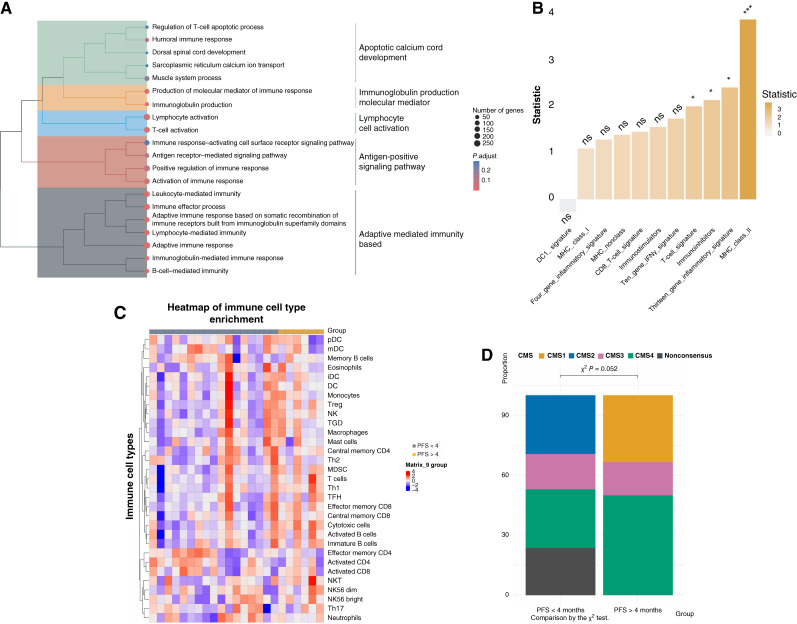
Gene expression signatures, immune cell enrichment and gene expression–based CMS, and association with treatment response. Treeplot depicting the results of hierarchical clustering of enriched terms from GSEA, comparing enriched pathways in patients with PFS <4 or >4 months (**A**). Bar plot reporting the results of the Student *t* test t statistics comparing inflammatory gene expression signature enrichment scores in patients with PFS >4 months vs. those with PFS <4 months (**B**). Heatmap of normalized enrichment scores of immune cell subtypes, comparing patients with PFS <4 or >4 months (**C**). DC, dendritic cells; iDC, immature dendritic cells; mDC, mature dendritic cells; MDSC, myeloid-derived suppressor cells; pDC, plasmacytoid dendritic cells; TFG, follicular helper T cells; TGD, gamma-delta T cells; Treg, regulatory T cells. Stacked barplots representing the proportion of patients assigned to gene expression CMS in patients with PFS >4 months (*n* = 6) and patients with PFS <4 months (*n* = 17; **D**).

RNA-seq was successful in 23/27 (85%) evaluable cases, including six of eight with PFS >4 months. After batch correction (Supplementary Fig. S3A and S3B), no single gene emerged as a relevant predictor of such response according to its differential expression (Supplementary Fig. S3C), but GSEA revealed a significant enrichment of lymphocyte-mediated, adaptive immune response pathways in patients with PFS >4 months (Fig. 2A; Supplementary Fig. S3D). Similarly, single-sample GSEA showed a significant enrichment in inflammatory pathways and immunomodulators, including MHC class II and T-cell signatures (Fig. 2B; Supplementary Table S2). Virtual microdissection of the tumor microenvironment confirmed significantly higher lymphocytic infiltration, mainly represented by Th1 and cytotoxic T lymphocytes, in patients with PFS >4 months (Fig. 2C; Supplementary Fig. S4; Supplementary Tables S3 and S4).

Analysis of colorectal cancer transcriptomic subtypes revealed that CMS1 was identified only among patients with PFS >4 months (who also had a higher relative proportion of the CMS4 subtype), whereas the CMS2 subtype was restricted to patients without a relevant clinical response (Fig. 2D; Supplementary Fig. S5A). A positive DetermaIO binary score (26) was more frequently observed in patients with PFS >4 months (33.3% vs. 5.9%; *P* = 0.086) and was associated with a longer median PFS (11.1 vs. 1.9 months; log-rank *P* = 0.054; Supplementary Fig. S5B). Interestingly, PD-L1 expression by CPS could be assessed in 25/27 patients and was a good predictor of response. In fact, two of the three patients with PR had CPS ≥5, and the proportion of patients with DCR almost doubled with CPS ≥5 (80% vs. 45%) and tended to be higher already with CPS ≥1 [Fig. 1D (waterfall plot); Table 4].

**Table 4.** ORR, DCR, and PFS >4 months according to PD-L1 CPS.

	CPS < 1	CPS ≥ 1	CPS < 5	CPS ≥ 5	Total
ORR, *n* (%)					
ORR (PR)	1 (6.7)	2 (20.0)	1 (5.0)	2 (40.0)	3
No ORR (SD + PD)^a^	14 (93.3)	8 (80.0)	19 (95.0)	3 (60.0)	22
Total	15	10	20	5	25
DCR, *n* (%)					
DCR (PR + SD)	7 (46.7)	6 (60.0)	9 (45.0)	4 (80.0)	13
No DCR (PD)	8 (53.3)	4 (40.0)	11 (55.0)	1 (20.0)	12
Total	15	10	20	5	25
PFS >4 months, *n* (%)					
Yes	2 (13.3)	5 (50.0)	4 (20.0)	3 (60.0)	7
No	13 (86.7)	5 (50.0)	16 (80.0)	2 (40.0)	18
Total	15	10	20	5	25

Abbreviation: PD, progressive disease.

a Patients who have not achieved CR or PR at any timepoint throughout the study.

Moreover, PFS >4 months occurred in a higher proportion of patients with CPS ≥1 (50% vs. 13%) and CPS ≥5 (60% vs. 20%; Table 4).

## Discussion

Vorbipiprant is a PGE2 EP4 receptor antagonist that specifically modulates the immune response within the tumor microenvironment (13–15). In the present phase Ib/IIa trial, we explored the potential of vorbipiprant to induce sensitization of pMMR/MSS mCRC to an anti–PD-1 antibody, namely, balstilimab. The study yielded several findings. First, vorbipiprant in combination with PD-1 blockade was well tolerated across dosages ranging from 30 to 180 mg twice daily. Second, the study achieved a DCR of 50% among all participants and more than 30% within each dose group, with an ORR of 11% in the entire cohort. Third, comprehensive molecular profiling identified possibly predictive biomarkers of efficacy in the absence of specific clinical characteristics associated with clinical benefit.

Numerous combination strategies are currently being investigated to improve the efficacy of immunotherapy in pMMR/MSS mCRC (27). These approaches encompass combinations of immunotherapy with chemotherapy, radiotherapy, targeted therapies, bispecific antibodies, cancer vaccines, or intratumoral therapies (27). Despite the innovative nature of these combinations, in most cases, the limited efficacy and/or the safety profile remains of paramount concern, with potential risk of severe, in some instances life-threatening, irAE.

Recently, the combination of balstilimab with botensilimab, an Fc-enhanced anti–CTLA-4 antibody, has demonstrated encouraging results in pMMR/MSS mCRC (17, 18), pending results of randomized trials. However, preliminary efficacy of this combination was restricted to patients with pMMR/MSS mCRC without liver metastases (18). Phase III trials evaluating PD-1/LAG-3 co-targeting with nivolumab/relatlimab or the combination of pembrolizumab with the multitargeted tyrosine kinase inhibitor lenvatinib produced negative results (28, 29), underscoring the pressing need for novel therapeutic options for this patient population.

The vorbipiprant safety profile in combination with a PD-1 inhibitor looks acceptable: No DLT were observed, and there were no safety concerns from TEAE that required permanent discontinuation of the study drugs. The single serious reaction of duodenal ulcer hemorrhage, prompting the use of proton pump inhibitors, is consistent with the role of EP4 receptors in modulating gastrointestinal mucosal integrity (30). This event is not an off-target toxicity and was observed with other EP4 antagonists (31), suggesting that this may be a class effect. Some patients experienced a vorbipiprant-related transient increase in alkaline phosphatase levels, with no accompanying signs or symptoms of cholestasis. This effect was already observed in previous vorbipiprant studies (14), was not deemed clinically relevant, and remains mechanistically unexplained. No safety concerns emerged from irAE. One serious irAE of pneumonitis was attributed to balstilimab and easily resolved with corticosteroids. Other irAE requiring medical treatment occurred in an additional five patients, thus overall affecting 21% of patients. Thanks to the bipartite functions of PGE2 via the EP4 receptor (immunosuppressor in the cancer microenvironment and immunostimulant in autoimmune diseases; ref. 32), vorbipiprant may have the additional theoretical advantage of counteracting the onset or progression of irAE induced by ICI: this hypothesis should be investigated in future studies.

In addition to demonstrating an acceptable safety profile, the trial provided preliminary evidence of efficacy. Besides DCR, supportive results were observed across secondary efficacy endpoints, including an ORR of 11% in the entire study cohort with three PR, all occurring in patients with TMB-low status. The median PFS may not fully capture the benefit of ICI; however, preliminary OS results were encouraging, despite the absence of a control group.

Patients with liver metastases were included in the present study, whereas they are currently excluded from studies of novel immunotherapy combinations (18) because of their typically poor prognostic impact (33, 34) and studies suggesting immune tolerance of liver metastases (35). Based on the preliminary results observed here, vorbipiprant plus anti–PD-1 agents, as a stand-alone combination or together with other drugs such as chemotherapy or anti-angiogenics, should be further investigated regardless of liver metastases and potentially using more accurate immune-related biomarkers.

Overall, these findings support further evaluation of vorbipiprant’s ability to immune-sensitize a subset of patients with pMMR/MSS mCRC to ICI. To this aim, we used longitudinally collected blood and tissue samples to predict benefit from vorbipiprant-based ICI combinations. We found that tumors with some degree of immune infiltration are more likely to benefit from this experimental strategy, including those showing noncanonical consensus molecular subtypes (i.e., non-CMS2). In fact, we showed more sustained clinical benefit in patients whose tumor tissues had T-lymphocyte enrichment and immune-enriched pathways. Analysis of the DetermaIO immune-oncology gene expression signature (26) showed that higher scores were associated with better clinical response. Finally, we also showed that PD-L1 expression assessed by CPS may serve as a surrogate of baseline immune-inflamed status and may be therefore an easy-to-assess, less expensive, and rapid assay to enrich patients with clinical benefit, given the complexity of RNA-seq or gene expression signatures. Also, from a pharmacodynamic point of view, we exploited liquid biopsies to noninvasively monitor and predict treatment benefit and showed that the immune-exhausted status at baseline may be reverted by T-cell activation in responders. As expected, a statistically significant increase in the T-cell–recruiting chemokine CXCL10 level was observed in patients after 2-month treatment, correlating with the increase in CD8^+^CD3^+^ cells.

Several clinical programs worldwide are investigating the combination of EP4 antagonists with ICI in patients with cold solid tumors, including MSS mCRC (36–38). Data from the present trial suggest that vorbipiprant may have a sufficient efficacy and good tolerability profile when combined with ICI because of its optimal pharmacologic and pharmacokinetic characteristics (13) and a large safety database in human studies (14).

Although this study is the first trial of vorbipiprant as a strategy to overcome resistance to ICI, several limitations are worth noting. The study nature and the limited number of participants may affect the precision of outcome measures and could potentially skew the observed safety and efficacy profiles, either underemphasizing or overemphasizing them. Second, the DCR was a co-primary endpoint of the study, and it is usually considered a weak surrogate endpoint for efficacy; therefore, the preliminary efficacy shown here on the more robust secondary endpoints must be confirmed and expanded in larger trials that may include an appropriate comparator arm.

Third, the 3 + 3 dose-finding design has limitations compared to other trial designs, which may be better suited to identify therapeutic signals typically observed in later stages of development (39). Nevertheless, careful dose expansion suggested no significant differences in safety or efficacy between the 30- and 90-mg twice-daily dosages, although the 90-mg cohort combined with the anti–PD-1 balstilimab had a slightly higher ORR (15%). Prior human data showed that the vorbipiprant 90-mg dose achieves higher calculated receptor occupancy than the 30-mg dose, potentially enabling better therapeutic exposure. Although the 180-mg twice-daily dosage was well tolerated and confirmed vorbipiprant’s safety margin, it did not confer additional benefits to the combination. This might be due to the smaller number of patients enrolled in this cohort, once it was understood that further benefit was unlikely by simply increasing the dose levels. We believe therefore sufficient dose optimization was achieved, and the 90-mg twice-daily dosage will be taken forward in future trials of combination with ICI. Another limitation is that the OS results may have been affected by postprogression treatments, received by approximately 70% of patients and by the selection of patients with a more indolent disease course. However, given that the patients in this study were heavily pretreated, the results are promising, especially considering the modest survival benefit typically offered by late-line treatments in this cohort.

Lastly, a more comprehensive understanding of response patterns and resistance mechanisms could be achieved by evaluating biomarkers at the start of study, during treatment, and at disease progression, as preliminarily suggested here. In this respect, a limitation of the translational analyses performed is the absence of data from posttreatment biopsies to analyze dynamic changes in the tumor immune contexture.

In conclusion, the present phase Ib/IIa trial showed that the combination of vorbipiprant and an anti–PD-1 (balstilimab) yielded responses in patients with chemorefractory, pMMR/MSS mCRC. Significantly, this combination demonstrated a favorable safety profile. Further studies are necessary to determine whether the observed beneficial effects lead to clinically meaningful long-term outcomes.

## Supplementary Material

Supplementary Methods S1 Supplementary Methods S1. PBMC analysis, Gene expression profiling, and Cytokines Measurement.

Supplementary Figure S1 Supplementary Figure S1. Kaplan Meier plot for PFS and OS overall and stratified by presence/absence of liver metastases.

Supplementary Figure S2 Supplementary Figure S2. PBMC subpopulation at baseline and at 2 months based on PFS duration or vorbipiprant dose.

Supplementary Figure S3 Supplementary Figure S3. RNA-seq analysis of gene expression and pathways signatures.

Supplementary Figure S4 Supplementary Figure S4. ESTIMATE-based deconvolution analysis comparing patients with PFS< or >4 months.

Supplementary Figure S5 Supplementary Figure S5. Analysis of the DetermaIO immune related gene signature.

Supplementary Table S1 Supplementary Table S1. Representativeness of Study Participants.

Supplementary Table S2 Supplementary Table S2. Logistic regression analysis results of inflammatory gene expression signatures enrichment scores as predictors of treatment response.

Supplementary Table S3 Supplementary Table S3. Results of Student's t-tests comparing enrichment scores of CIBERSORTx-based immune cell populations in patients with PFS< or >4months.

Supplementary Table S4 Supplementary Table S4. Logistic regression analysis results of immunecell phenotypes enrichment scores as predictors of treatment response.
