# Supplementary material for: The Prostaglandin EP4 Antagonist Vorbipiprant Combined with PD-1 Blockade for Refractory Microsatellite-Stable Metastatic Colorectal Cancer: A Phase Ib/IIa Trial
Source: Clin Cancer Res. 2024 Dec 2;31(4):649–58. doi: 10.1158/1078-0432.CCR-24-2611 (PMC11831105; doi:10.1158/1078-0432.CCR-24-2611)
Supplement: Supplementary Methods S1 — PBMC analysis, Gene expression profiling, and Cytokines Measurement. [file ccr-24-2611_supplementary_methods_s1_suppsm1.pdf]

Supplementary Methods

PBMC analysis

Frozen peripheral blood mononuclear cells (PBMCs) were thawed and tested by multi-color flow cytometry analysis to assess different immune cell populations. For intracellular markers, cells were fixed and permeabilized using the Foxp3 Transcription Factor Staining Buffer Set (eBioscience, Thermo Fisher) following manufacturer's protocol. The full antibodies list is reported below:

Fluorochrome	Marker	Clone	identifier	Supplier	RRID
BB515	ICOS (CD278)	DX29	564549	BD	AB_2738840
PE-CF594	CCR7 (CD197)	2-L1-A	566768	BD	AB_2869857
APC-R700	CD25	2A3	565106	BD	AB_2744339
BV421	Ki67	B56	562899	BD	AB_2686897
BV510	CD73	AD2	563198	BD	AB_2738062
BV 786	PD-1	MIH4	741018	BD	AB_2740639
PE-CF594	CD56	B159	562289	BD	AB_11152080
APC	PD-1 (CD279)	MIH4	558694	BD	AB_1645458
APC-H7	CD4	RPA-T4	560158	BD	AB_1645478
BV421	Granzyme B	GB11	563389	BD	AB_2738175
BV510	CD3	HIT3a	564713	BD	AB_2738909
BV 605	CD8	SK1	564116	BD	AB_2869551
FITC	CD3	SK7	345763	BD	AB_2811220
FITC	CD19	SJ25C1	345788	BD	AB_2868814
FITC	CD20	L27	345792	BD	AB_2868818
FITC	CD56	NCAM16.2	345811	BD	AB_2868832
PE	CD10	/	555375	BD	AB_395776
PE-CF594	CD11b	ICRF44	562399	BD	AB_2737613
BB700	CD16	RG8	746199	BD	AB_2743545
APC	HLADR	/	559866	BD	AB_398674
APC-R700	PD-L1	MIH1	565188	BD	AB_2869928
BV421	CD66b	G10F5	562940	BD	AB_2737906
BV510	CD14	M5E2	740163	BD	AB_2739916
BV 605	CD33	HIM3-4	744352	BD	AB_2742171
BV 786	CD15	HI98	563838	BD	AB_2738444
Alexa fluor 488	TCF7/TCF1	S33-966	567018	BD	AB_2916388
APC-H7	CD45RA	HI100	560674	BD	AB_1727497
BV 786	TIM 3	7D3	742857	BD	AB_2741100
APC	CD39	eBioA1	17-0399-42	eBioscience	AB_10804519
PerCP E-fluor 710	Ki67	20RaJ1	46-5699-42	eBioscience	AB_10804653
PE	FOXP3	PCH 101	12-4776-42	eBioscience	AB_1518782

Samples were acquired using a Celesta BD Cytometer equipped with BD FACSDiva software and analysis performed using FlowJo V10 software (RRID:SCR_008520). The abundance of peripheral blood mononuclear cells (PBMCs) subpopulations at baseline was expressed as the relative fraction within 5 major leukocytes subtype (CD8+ lymphocytes, NK cells, conventional CD4

lymphocytes, T regulatory (Treg) lymphocytes and Myeloid Cells). The variation between the baseline and the subsequent timepoints was expressed as a relative percent change from the baseline abundance. Differences in the enrichment of baseline PBMCs subpopulations and their variations across timepoints were modelled with the Wilcoxon signed-rank test, the respective p values were corrected with the Benjamini–Hochberg procedure and considered significant below a threshold of 0.10.

Gene expression profiling

RNA sequencing (RNA-seq) was performed on a subset of FFPE tumor samples available at baseline. Total RNA was extracted using QIAGEN RNeasy FFPE Kit, followed by library preparation with Illumina TruSeq Stranded Total RNA Gold. Sequencing was performed on the Illumina Novoseq platform to a read depth of 50 million reads, generating 150-bp paired-end reads. The resulting sequenced reads were aligned to the reference genome using Spliced Transcripts Alignment to a Reference (STAR) (RRID:SCR_004463). Gene expression was calculated using the RSEM[1] package (Ensembl release 75) v1.3.3 and Ensembl GRCh38 release 75 gene annotation (RRID:SCR_002344). Ensembl versioned gene identifiers were converted to Hugo (RRID:SCR_012800) Symbols and Entrez (RRID:SCR_016640) identifiers using the BiomaRt package (RRID:SCR_019214).[2] Count data were explored according to the standard DESeq2 (RRID:SCR_000154) ipeline.[3] Data were normalized using variance stabilizing transformation (VST), and were subsequently used to obtain the 27-gene DetermaIO score, as previously described,[4] with an additional threshold for positivity as proposed in the AtezoTRIBE translational study.[5] Moreover, principal component analysis (PCA) was performed on VST transformed data, highlighting significant clustering of samples according to the tissue of origin of each biopsy. Batch correction according to the clustered biopsy site (liver vs lung vs gastrointestinal tract biopsies) was performed on count data and on TPMs by means of surrogate variable analysis,[6] and corrected data were used for downstream analyses. Differential expression analysis was performed using DESeq2 and the resulting log-fold change values were used as inputs for GSEA using the Gene Ontology (GO) Biological Processes resource with the clusterProfiler[7] package.

A curated list of inflammatory gene expression signatures was used as input to perform a single-sample GSEA (ssGSEA) and obtain enrichment scores of each pathway for each case using the GSVA[8] package, and the predictive role of each signature on treatment response was assessed by means of logistic regression analysis.

Deconvolution methods were applied to virtually microdissect the tumor microenvironment and evaluate the differential enrichment of the immune compartment and of individual immune cell subpopulations according to tumor response. In detail, the ESTIMATE[9] algorithm was used to derive an immune score for each sample, while CIBERSORTx[10] was adopted to infer enrichment of immune cell subtypes. In addition, given the lack of specific immune cell subtypes (e.g. myeloid-derived suppressor cells, MDSC) profiles in the above mentioned methods, ssGSEA was also applied using a curated list of genes related to tumor-infiltrating lymphocyte subpopulations, as previously defined,[11] and differential enrichment was assessed by means of logistic regression.

Consensus molecular subtypes were defined by means of consensus clustering based on the original classifier genes,[12] followed by semi-automatic subtype assignment based on gene expression patterns via the Cola package (RRID:SCR_006442). [13]

Plots and graphs were generated using the ggplot2 (RRID:SCR_014601), ComplexHeatmap (RRID:SCR_017270)[14] and enrichplot packages.

Cytokines Measurement

The plasma levels of CXCL10, IL-10, CCL5, IL-12 and IFN-γ was determined using commercially available ELISA kits (Quantikine®ELISA R&D Systems Inc., Minneapolis, MN, USA), according to manufacturer's protocols: DIP100 Human CXCL10/IP-10 Quantikine ELISA Kit, D1000B Human IL-10 Quantikine ELISA Kit, D1200 Human IL-12 Quantikine ELISA Kit, DRN00B Human CCL5/RANTES Quantikine ELISA Kit, DIF50C Human IFN-gamma Quantikine ELISA Kit. The optical density of the wells was determined with a microplate reader (iMark Microplate Absorbance Reader, Biorad, Hercules, CA, USA) set to 450 nm.

References

1. Li B, Dewey CN.

   RSEM: accurate transcript quantification from RNA-Seq data with or without a reference genome.

   BMC Bioinformatics **2011**;12:323. doi: 10.1186/1471-2105-12-323

2. Durinck S, Spellman PT, Birney E, Huber W.

   Mapping Identifiers for the integration of Genomic Datasets with the R/Bioconductor package biomaRt.

   Nat Protoc **2009**;4(8):1184-1191. doi:10.1038/nprot.2009.97.

3. Love MI, Huber W, Anders S.

   Moderated estimation of fold change and dispersion for RNA-seq data with DESeq2.

   Genome Biol **2014**;15:550. doi:10.1186/s13059-014-0550-8.

4. Nielsen TJ, Ring BZ, Seitz RS, Hout DR, Schweitzer BL.

   A novel immuno-oncology algorithm measuring tumor microenvironment to predict response to immunotherapies.

   Heliyon **2021**;7(3):e06438. doi.org/10.1016/j.heliyon.2021.e06438

5. Antoniotti C, Boccaccino A, Seitz R, Giordano M, Catteau A, Rossini D, *et al.*

   An Immune-Related Gene Expression Signature Predicts Benefit from Adding Atezolizumab to FOLFOXIRI plus Bevacizumab in Metastatic Colorectal.

   Clin Cancer Res **2023**;29(12):2291–2298. doi.org/10.1158/1078-0432.CCR-22-3878

6. Leek JT, Johnson WE, Parker HS, Jaffe AE, Storey JD.

The sva package for removing batch effects and other unwanted variation in high-throughput experiments.

Bioinformatics **2012**;28(6):882-883.

7. Yu G, Wang LG, Han Y, He QY.

clusterProfiler: an R package for comparing biological themes among gene clusters.

OMICS **2012**;16(5):284-287.

8. Hanzelmann S, Castelo R, Guinney J.

GSVA: gene set variation analysis for microarray and RNA-seq data.

BMC Bioinformatics **2013**;14:7. http://www.biomedcentral.com/1471-2105/14/7

9. Yoshihara K, Shahmoradgoli M, Martinez E, Vegesna R, Kim H, Torres-Garcia W, *et al.*

Inferring tumour purity and stromal and immune cell admixture from expression data.

Nat Commun **2013**;4:2612. doi: 10.1038/ncomms3612

10. Newman AM, Steen CB, Liu CL, Gentles AJ, Chaudhuri AA, Scherer F, *et al.*

Determining cell-type abundance and expression from bulk tissues with digital cytometry.

Nat Biotechnol **2019**;37(7):773-782. doi:10.1038/s41587-019-0114-2.

11. Angelova M, Charoentong P, Hackl H Fischer M.

Characterization of the immunophenotypes and antigenomes of colorectal cancers reveals distinct tumor escape mechanisms and novel targets for immunotherapy.

Genome Biol **2015**;16(1):64. DOI 10.1186/s13059-015-0620-6

12. Guinney J, Dienstmann R, Wang X, de Reyniès A, Schlicker A, Soneson C, *et al.*

The Consensus Molecular Subtypes of Colorectal Cancer.

Nat Med **2015**;21(11):1350-1356. doi: 10.1038/nm.3967.

13. Gu Z, Schlesner M, Hubschmann D.

cola: an R/Bioconductor package for consensus partitioning through a general framework.

Nucleic Acids Res **2021**;49(3):e15. doi: 10.1093/nar/gkaa1146

14. Gu Z, Eils R, Schlesner M.

Complex heatmaps reveal patterns and correlations in multidimensional genomic data. *Bioinformatics* **2016**;32(18):2847-2849.
