# Supplementary material for: The Prostaglandin EP4 Antagonist Vorbipiprant Combined with PD-1 Blockade for Refractory Microsatellite-Stable Metastatic Colorectal Cancer: A Phase Ib/IIa Trial
Source: Clin Cancer Res. 2024 Dec 2;31(4):649–58. doi: 10.1158/1078-0432.CCR-24-2611 (PMC11831105; doi:10.1158/1078-0432.CCR-24-2611)
Supplement: Supplementary Figure S1 — Kaplan Meier plot for PFS and OS overall and stratified by presence/absence of liver metastases. [file ccr-24-2611_supplementary_figure_s1_suppsf1.pdf]

**Supplementary Figure S1. Kaplan Meier plot for PFS and OS overall (panels A, B) and stratified by presence/absence of liver metastases (panels C, D).**

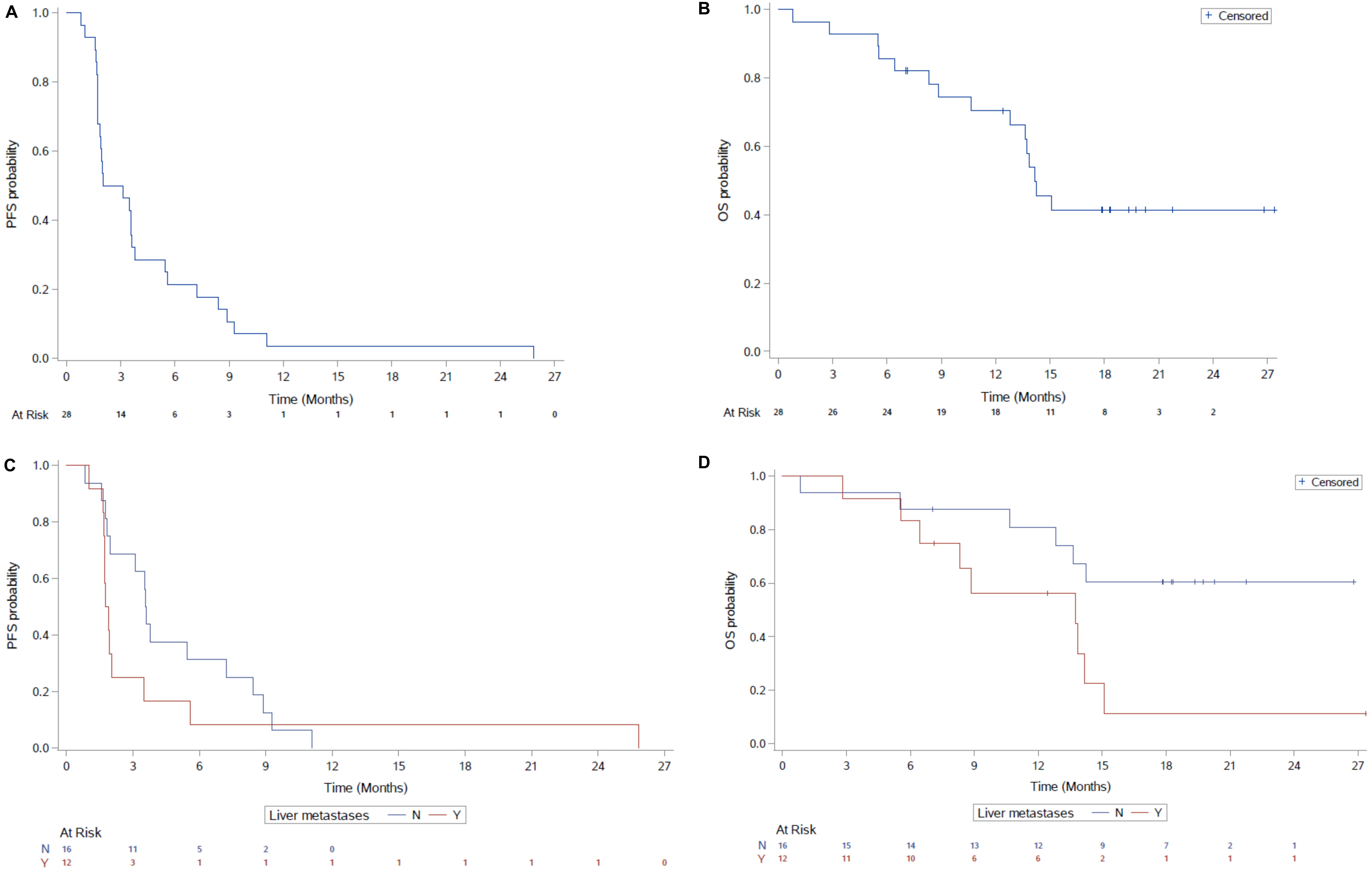


Median follow-up in the entire cohort (n=28, panels A, B) was 13.8 months (IQR: 7.7-18.3).
Median follow-up in patients with or without liver metastases was 10.6 months (IQR: 6.8-14.0) and 17.9 months (IQR:11.7-19.5)
