# Supplementary material for: The Prostaglandin EP4 Antagonist Vorbipiprant Combined with PD-1 Blockade for Refractory Microsatellite-Stable Metastatic Colorectal Cancer: A Phase Ib/IIa Trial
Source: Clin Cancer Res. 2024 Dec 2;31(4):649–58. doi: 10.1158/1078-0432.CCR-24-2611 (PMC11831105; doi:10.1158/1078-0432.CCR-24-2611)
Supplement: Supplementary Figure S2 — PBMC subpopulation at baseline and at 2 months based on PFS duration or vorbipiprant dose. [file ccr-24-2611_supplementary_figure_s2_suppsf2.pdf]

Supplementary Figure S2. PBMC subpopulations at baseline and at the 2-month early endpoint based on PFS duration (< or >4 months) or vorbipiprant dose, with changes in selected cytokines.

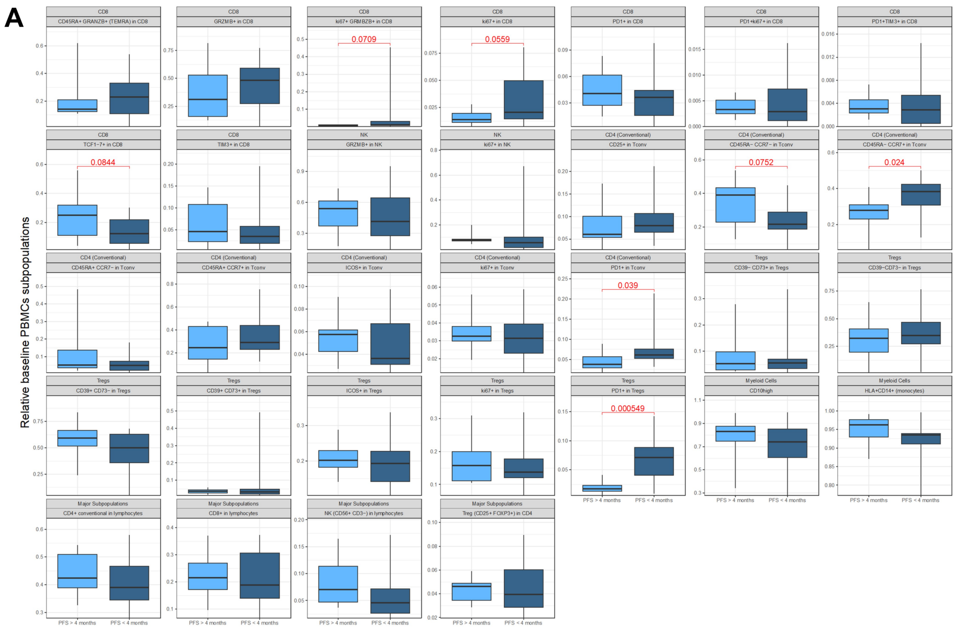

Wilcoxon signed-rank test was used to model differences in the relative abundance in patients with PFS< or >4 months; p values were corrected with the Benjamini–Hochberg procedure

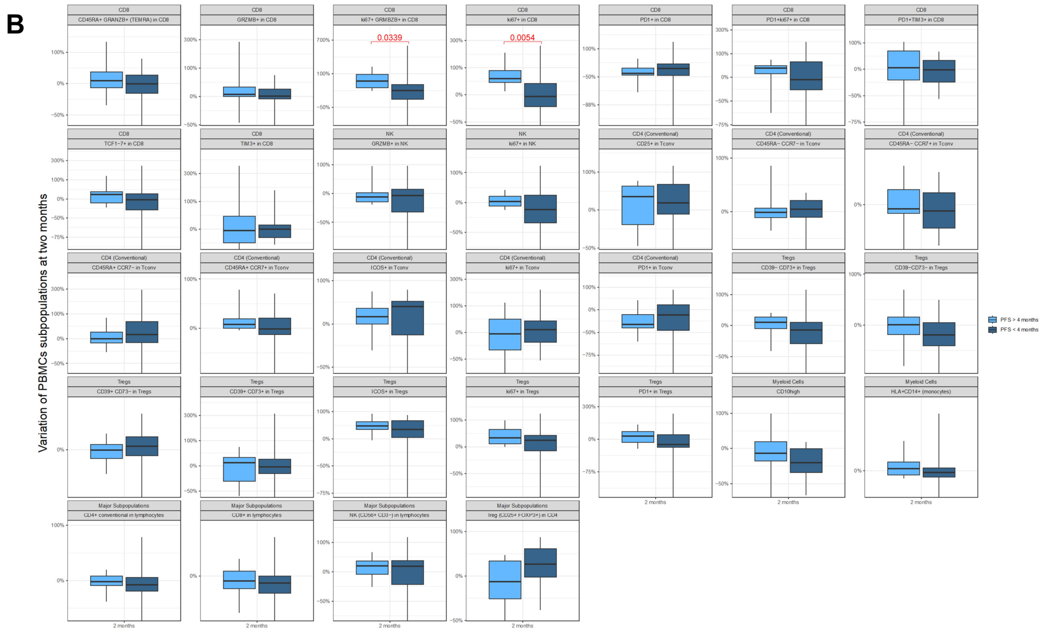

Wilcoxon signed-rank test was used to model differences in the relative abundance, p values were corrected with the Benjamini–Hochberg procedure.

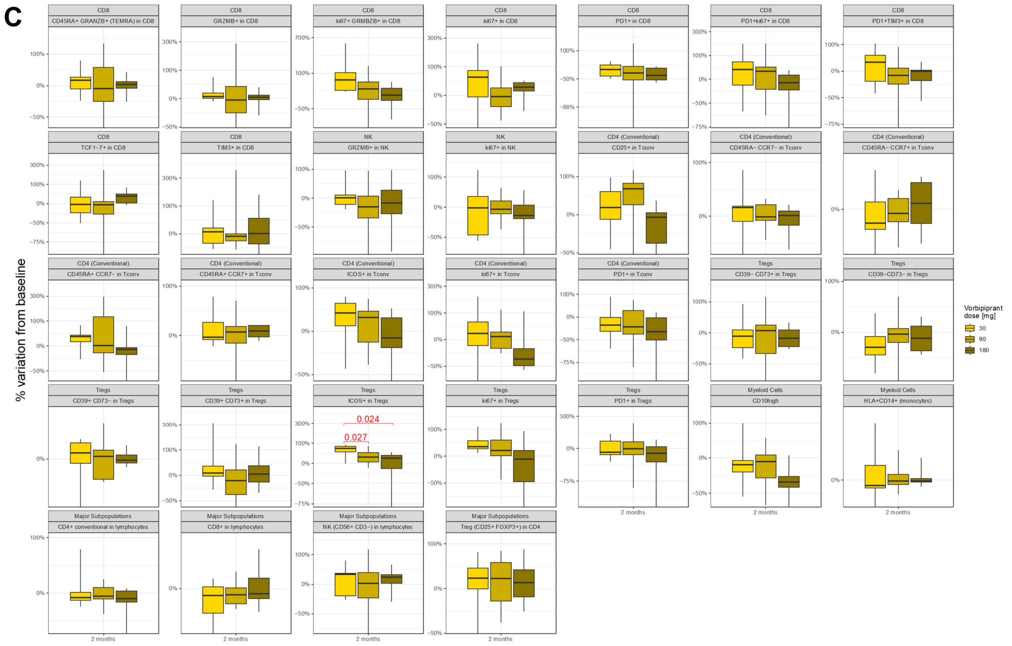

Wilcoxon signed-rank test was used to model differences in the relative abundance, p values were corrected with the Benjamini–Hochberg procedure

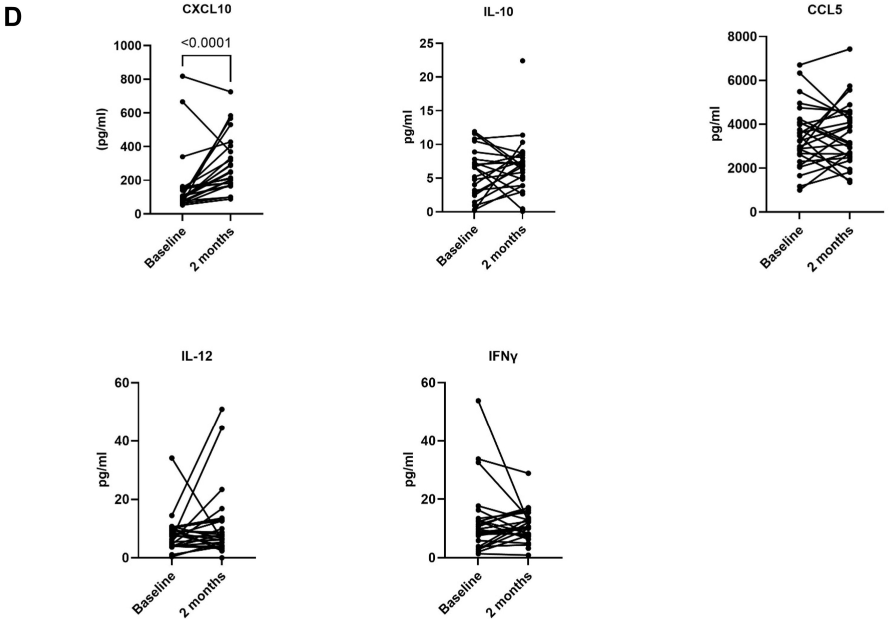

Wilcoxon signed rank test was used for statistical calculation.

(A) Relative abundance of baseline PBMC subpopulations at baseline. **(B and C)** Variation of PBMCs subpopulations at 2 months, expressed as % variation from baseline, depending **(B)** on PFS duration (< or >4 months) or on the vorbipiprant dose **(C)**. **(D)** Changes in serum levels of CXCL10, IL-10, CCL5, IL-12, IFNγ from baseline to 2 months.
