# Supplementary material for: The Prostaglandin EP4 Antagonist Vorbipiprant Combined with PD-1 Blockade for Refractory Microsatellite-Stable Metastatic Colorectal Cancer: A Phase Ib/IIa Trial
Source: Clin Cancer Res. 2024 Dec 2;31(4):649–58. doi: 10.1158/1078-0432.CCR-24-2611 (PMC11831105; doi:10.1158/1078-0432.CCR-24-2611)
Supplement: Supplementary Figure S3 — RNA-seq analysis of gene expression and pathways signatures. [file ccr-24-2611_supplementary_figure_s3_suppsf3.pdf]

# Supplementary Figure S3. RNA-seq analysis of gene expression and pathway signatures

**A**

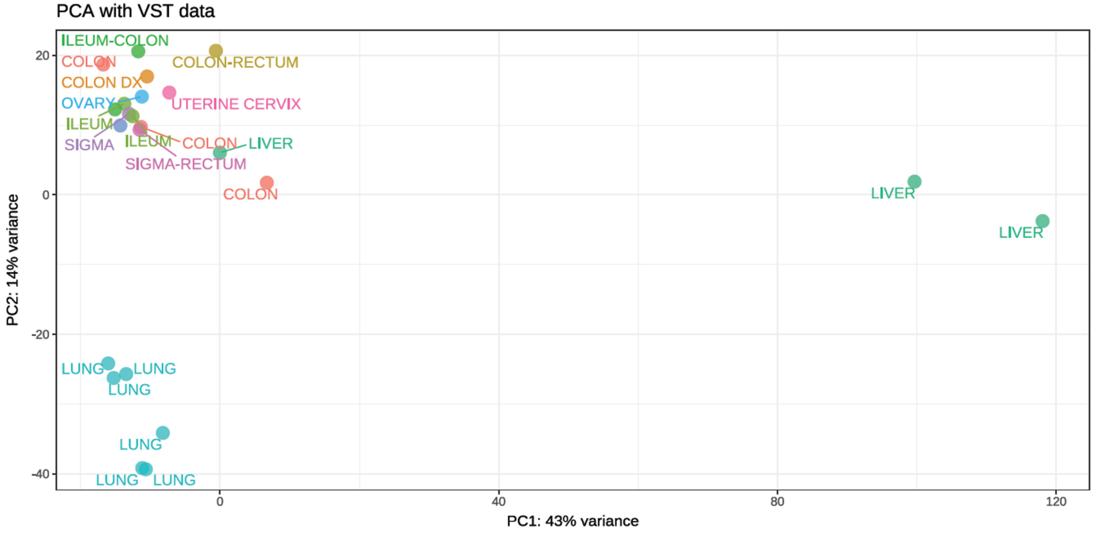


Each point represents a patient/tumor sample and is coloured according to the tissue of origin

**B**

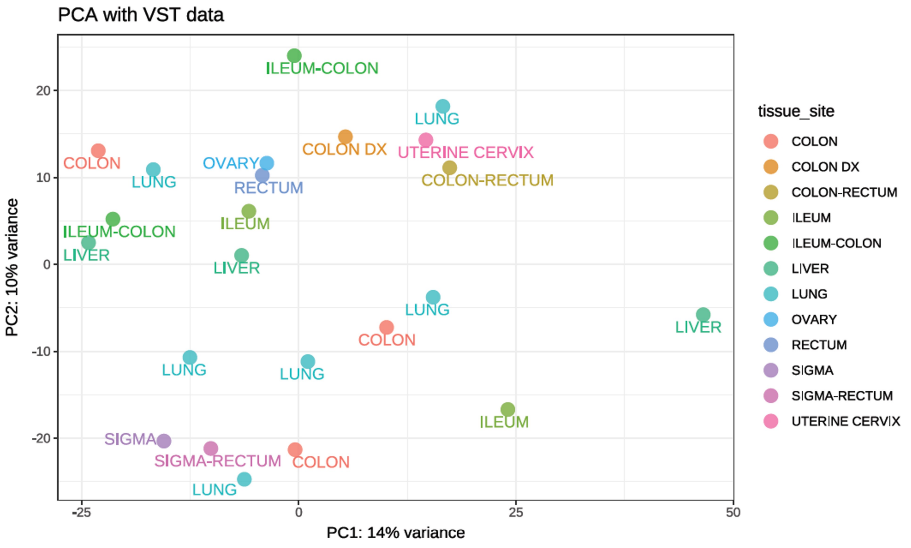


Each point represents a patient/tumor sample and is coloured according to the tissue of origin

**C**

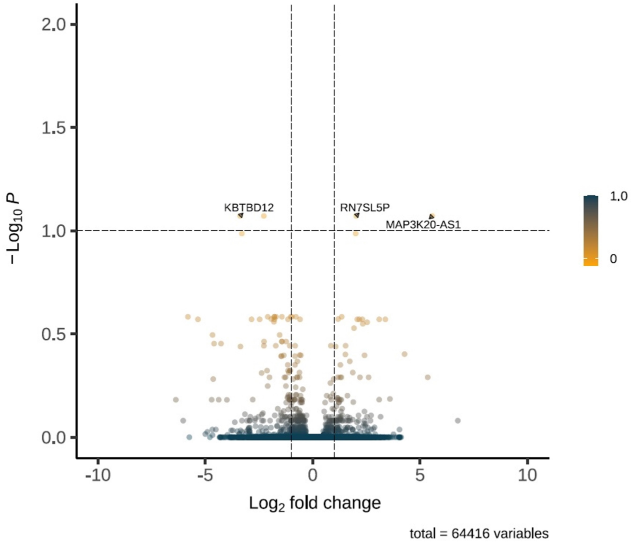


The dotted lines are plotted vertically at an absolute log2 fold change = 1 and horizontally at an adjusted p value = 0.1, corresponding to -Log10(0.1) = 1. Points are coloured according to significance, and genes with adjusted p values < 0.1 are annotated with Hugo Symbols, when known.

**D**

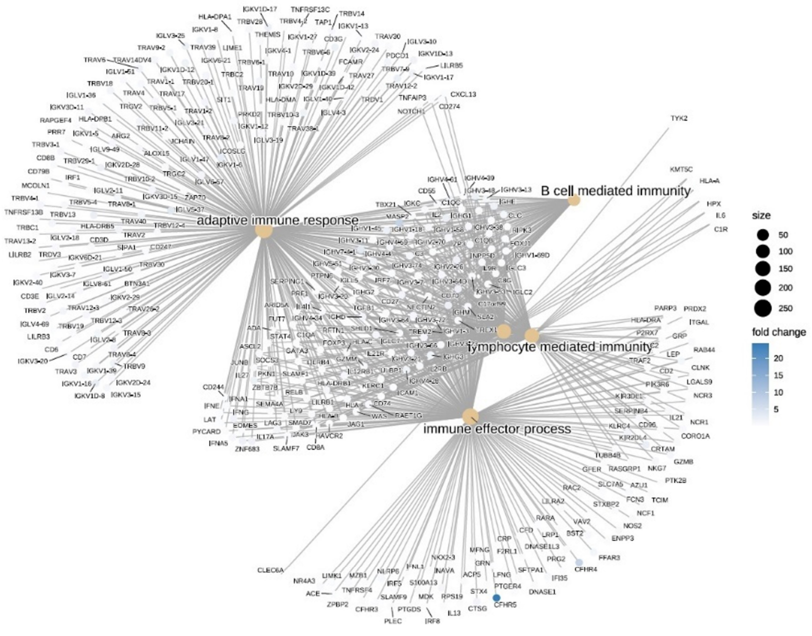


Each spoke is coloured according to the fold change in responders as compared to non-responders, and each hub size is plotted according to the number of related genes.

**(A and B)** Principal component analysis (PCA) of variance-stabilizing-transformed (VST) gene expression data before **(A)** and after **(B)** tissue-based batch correction. **(C)** Volcano plot of differential expression analysis results comparing patients with PFS < or >4 months. **(D)** Network plot reporting gene linkages of genes and biological pathways enriched in patients with PFS>4 months as compared to patients with PFS<4 months.
