# Supplementary material for: The Prostaglandin EP4 Antagonist Vorbipiprant Combined with PD-1 Blockade for Refractory Microsatellite-Stable Metastatic Colorectal Cancer: A Phase Ib/IIa Trial
Source: Clin Cancer Res. 2024 Dec 2;31(4):649–58. doi: 10.1158/1078-0432.CCR-24-2611 (PMC11831105; doi:10.1158/1078-0432.CCR-24-2611)

Supplementary Figure S4. ESTIMATE-based deconvolution analysis comparing patients with PFS< or >4 months.
Comparison of ESTIMATE Score, Immune Score, Stromal Score and Tumor Purity in patients with PFS <4 and PFS >4 months

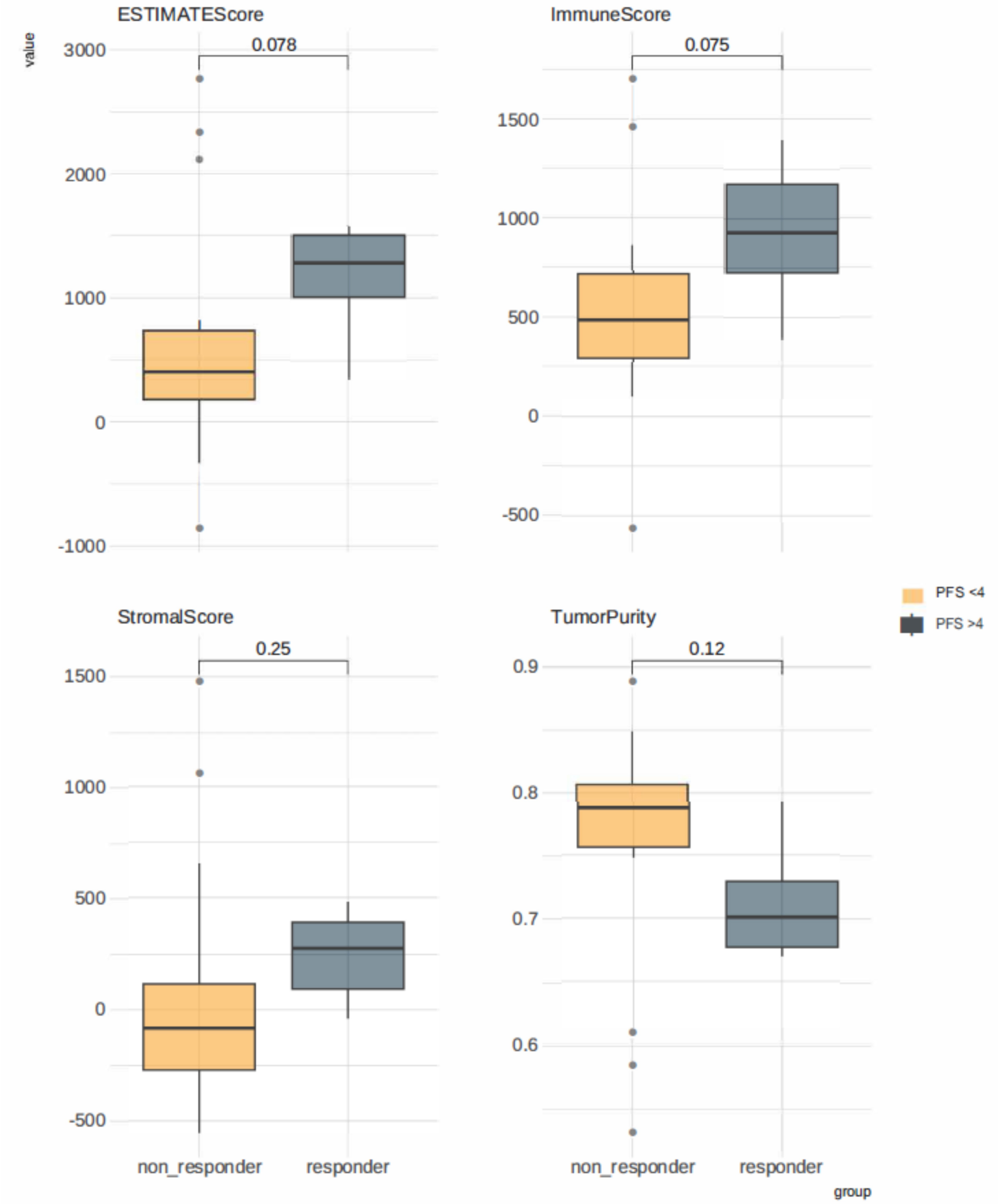

Supplement: Supplementary Figure S4 — ESTIMATE-based deconvolution analysis comparing patients with PFS< or >4 months. [file ccr-24-2611_supplementary_figure_s4_suppsf4.pdf]
