# Supplementary material for: The Prostaglandin EP4 Antagonist Vorbipiprant Combined with PD-1 Blockade for Refractory Microsatellite-Stable Metastatic Colorectal Cancer: A Phase Ib/IIa Trial
Source: Clin Cancer Res. 2024 Dec 2;31(4):649–58. doi: 10.1158/1078-0432.CCR-24-2611 (PMC11831105; doi:10.1158/1078-0432.CCR-24-2611)
Supplement: Supplementary Figure S5 — Analysis of the DetermaIO immune related gene signature. [file ccr-24-2611_supplementary_figure_s5_suppsf5.pdf]

Supplementary Figure S5. Analysis of the DetermaIO immune-related gene signature

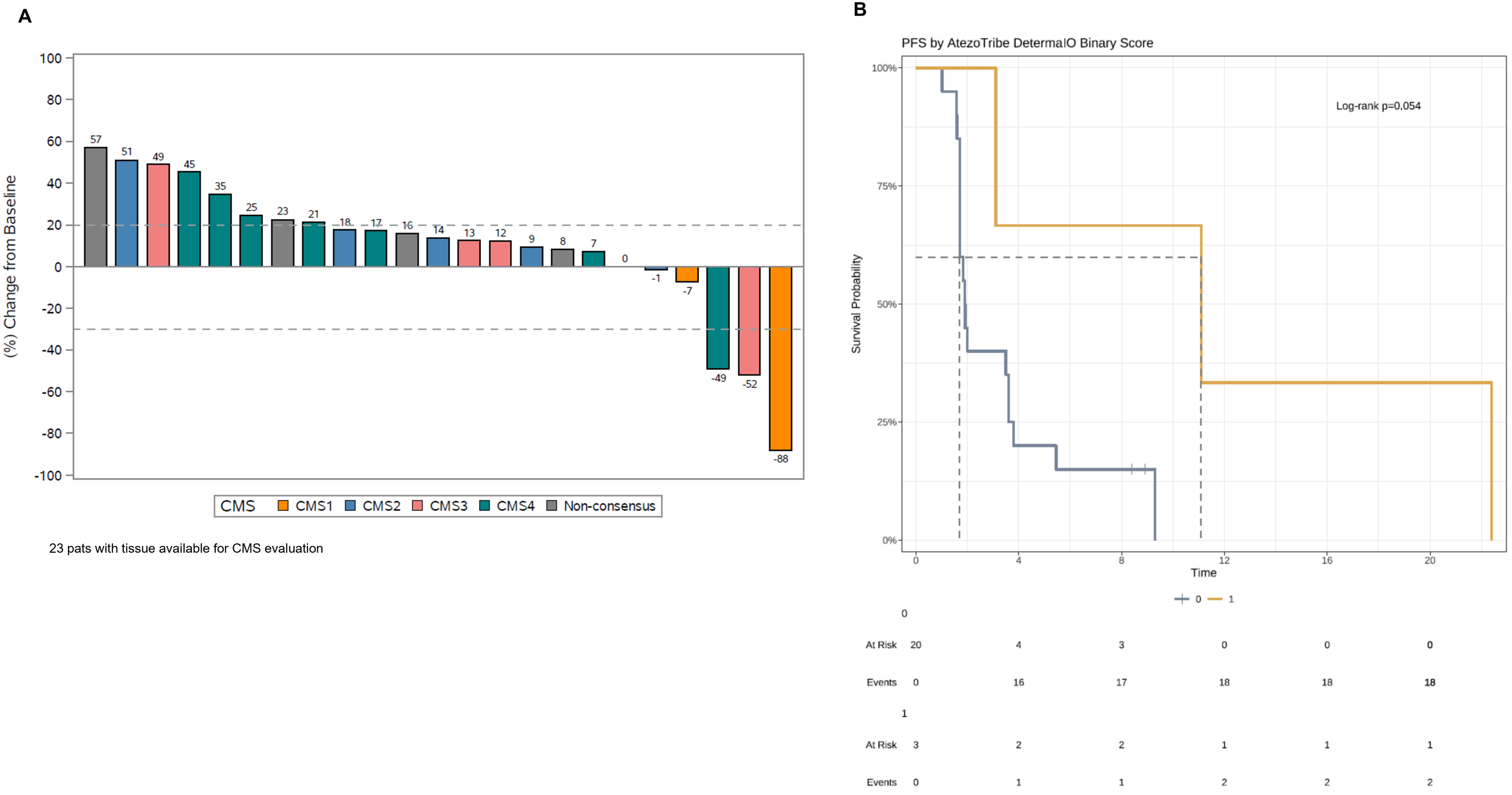

(A) Waterfall plot reporting treatment response according to consensus molecular subtypes (CMS). **(B)** Kaplan Meier plot for PFS < or > 4 months stratified by the dichotomized DetermaIO score.
