# Supplementary material for: The Prostaglandin EP4 Antagonist Vorbipiprant Combined with PD-1 Blockade for Refractory Microsatellite-Stable Metastatic Colorectal Cancer: A Phase Ib/IIa Trial
Source: Clin Cancer Res. 2024 Dec 2;31(4):649–58. doi: 10.1158/1078-0432.CCR-24-2611 (PMC11831105; doi:10.1158/1078-0432.CCR-24-2611)
Supplement: Supplementary Table S1 — Representativeness of Study Participants. [file ccr-24-2611_supplementary_table_s1_suppst1.pdf]

Supplementary Table S1. Representativeness of Study Participants

Cancer type: Colorectal cancer (CRC)	
Considerations related to:	
Sex	More men than women are affected by and die of CRC. In the United States (USA), the incidence of CRC is approximately 44/100.000 males and 34/100.000 females, with mortality of approximately 17/100.000 males and 12/100.000 females[1]. In Europe, CRC is the third most diagnosed cancer in men (after prostate and lung cancers) and the second one in women (after breast cancer). It is the second cause of cancer death in men (after lung cancer) and the third one in women (after breast and lung cancers)[2].
Age	Most cases of CRC occur in people older than 45 years. The median age at diagnosis is 66 years in men and 69 years in women. Although the risk for CRC increases with age, there is a trend for a decrease in the age at diagnosis[1,2].
Race/ethnicity	CRC incidence and mortality vary by race/ethnicity: in the USA, it is highest in Black people and lowest in Asians, with Whites (the majority of the population in Europe) in between[3].
Geography	The incidence of CRC is similar in the USA and in Europe. However, mortality is higher in Europe, particularly in Central and Eastern Europe compared with Western Europe[3].
Other considerations	This study was performed in patients with microsatellite stable/proficient mismatch repair tumors, that represent approximately 95% of patients with metastatic CRC (mCRC) and typically do not respond to immunotherapy.
Overall representativeness of this study	This study is representative of the chemorefractory mCRC population in Western Europe, where the study was performed (Italy). As in the general CRC population, there were more men (54%) than women (46%), and all of them (100%) were Whites. The median age was 59 years, i.e. younger than the median age at diagnosis of CRC, possibly because of the required ECOG performance status 0-1. Active liver metastases were present in 43% patients.

References:

1. Colorectal Cancer Facts & Figures 2020-2022, American Cancer Society
2. Colorectal cancer burden in EU-27, European Cancer Information System (eCIS)
3. GBD 2019 Colorectal Cancer Collaborators. Global, regional, and national burden of colorectal cancer and its risk factors, 1990-2019: a systematic analysis for the Global Burden of Disease Study 2019. Lancet Gastroenterol Hepatol. 2022;7:627-47. doi: 10.1016/S2468-1253(22)00044-9.
