# Supplementary material for: The Prostaglandin EP4 Antagonist Vorbipiprant Combined with PD-1 Blockade for Refractory Microsatellite-Stable Metastatic Colorectal Cancer: A Phase Ib/IIa Trial
Source: Clin Cancer Res. 2024 Dec 2;31(4):649–58. doi: 10.1158/1078-0432.CCR-24-2611 (PMC11831105; doi:10.1158/1078-0432.CCR-24-2611)
Supplement: Supplementary Table S2 — Logistic regression analysis results of inflammatory gene expression signatures enrichment scores as predictors of treatment response. [file ccr-24-2611_supplementary_table_s2_suppst2.pdf]

Supplementary Table S2. Logistic regression analysis results of inflammatory gene expression signatures enrichment scores as predictors of treatment response.

Signature	Genes included in signature	Odds ratio	conf.low	conf.high	p.value
13-gene inflammatory signature	CCL2, CCL3, CCL4, CD8A, CXCL9, CXCL10, GZMK, HLA-DMA, HLA-DMB, HLA-DOA, HLA-DOB, ICOS, IRF1	4,03	1,31	18,96	0,033
MHC Class II signature	DRB6, DQB2, DPB2, DRB1, DBB5, DQA1, DQA2, DQB1, DPA1, DPB1	9,32	1,68	169,77	0,047
10-gene IFNg signature	CCR5, CXCL9, CXCL10, CXCL11, GZMA, HLA-DRA, IDO1, IFNG, PRF1, STAT1	3,24	1,17	13,87	0,048
Immunoinhibitors signature	CTLA4, PD1, IDO1, TGFb, TGFbR, BTLA, CD160, PD−L1, LAG3, PD−L2, HVEM, IL−10, IL−10Rb	4,02	1,26	22,64	0,051
T-cell signature	CD2, CD3D, CD3E	4,20	1,21	31,83	0,076
Immunostimulators signature	B7−1, B7H2, ICOS, CD137L, CD137, OX40, OX40L, CD70, GITRL, IL−6, STING, CD27, TACI, BCMA, IL−6R, B7−2, CD28, GITR, BAFF, BAFFR, APRIL, CD40L, CD40	2,45	0,84	9,60	0,139
4-gene inflammatory signature	CD274 (PD-L1), CD8A, LAG3, STAT1	2,36	0,89	9,82	0,139
CD8 T-cell signature	CD8A, CD8B	2,68	0,92	15,47	0,139
MHC non-class signature	HLA−G, HLA−E, HLA−F	1,98	0,75	6,11	0,186
MHC Class I signature	HLA−A, TAP1, TAP2, HLA−B, HLA−C, B2M	1,86	0,70	5,72	0,230
DC1 signature	CLEC9A, FLT3, XCR1	0,88	0,33	2,37	0,784

Abbreviations: conf.low/high: low/high confidence interval limit.
