# Supplementary material for: The Prostaglandin EP4 Antagonist Vorbipiprant Combined with PD-1 Blockade for Refractory Microsatellite-Stable Metastatic Colorectal Cancer: A Phase Ib/IIa Trial
Source: Clin Cancer Res. 2024 Dec 2;31(4):649–58. doi: 10.1158/1078-0432.CCR-24-2611 (PMC11831105; doi:10.1158/1078-0432.CCR-24-2611)
Supplement: Supplementary Table S3 — Results of Student's t-tests comparing enrichment scores of CIBERSORTx-based immune cell populations in patients with PFS< or >4months. [file ccr-24-2611_supplementary_table_s3_suppst3.pdf]

Supplementary Table S3. Results of Student's t-tests comparing enrichment scores of CIBERSORTx-based immune cell populations in patients with PFS< or >4months.

Immune cell population	statistic	df	p.value	p.adj
Mast cells activated	2,150	20,576	0,044	0,591
Dendritic cells activated	1,795	20,108	0,088	0,591
Macrophages M2	1,853	11,988	0,089	0,591
Dendritic cells resting	1,454	16,000	0,165	0,610
T cells follicular helper	-1,547	5,489	0,177	0,610
Neutrophils	1,377	20,598	0,183	0,610
Macrophages M1	-1,376	5,456	0,223	0,618
Plasma cells	-1,209	13,754	0,247	0,618
T cells CD4 naive	1,091	20,407	0,288	0,640
Macrophages M0	-0,945	6,759	0,377	0,650
T cells CD4 memory activated	-0,944	6,499	0,379	0,650
B cells naive	0,817	15,430	0,426	0,650
T cells CD8	-0,817	5,796	0,446	0,650
NK cells activated	0,763	18,929	0,455	0,650
Monocytes	0,615	20,537	0,545	0,716
Mast cells resting	0,574	19,480	0,573	0,716
B cells memory	-0,397	8,088	0,702	0,820
T cells CD4 memory resting	0,344	9,806	0,738	0,820
NK cells resting	0,227	16,064	0,823	0,841
Eosinophils	0,203	20,980	0,841	0,841

Abbreviations: df: degrees of freedom; p.adj: adjusted p value.
