# Supplementary material for: The Prostaglandin EP4 Antagonist Vorbipiprant Combined with PD-1 Blockade for Refractory Microsatellite-Stable Metastatic Colorectal Cancer: A Phase Ib/IIa Trial
Source: Clin Cancer Res. 2024 Dec 2;31(4):649–58. doi: 10.1158/1078-0432.CCR-24-2611 (PMC11831105; doi:10.1158/1078-0432.CCR-24-2611)
Supplement: Supplementary Table S4 — Logistic regression analysis results of immunecell phenotypes enrichment scores as predictors of treatment response. [file ccr-24-2611_supplementary_table_s4_suppst4.pdf]

Supplementary Table S4. Logistic regression analysis results of immune cell phenotypes enrichment scores as predictors of treatment response.

Immune Cell population	Gene	Odds Ratio	conf.low	conf.high	p.value
Activated B cells	AKNA, ARHGAP25, CCL21, CD180, CD2, CD27, CD37, CD38, CLEC17A, CLEC9A, CLECL1, FAIM3, FAM65B, GIMAP4, MAP4K1, PAX5, TNFRSF17, TRAF3IP3	2,61	0,86	11,78	0,140
Activated CD4	ANLN, BRIP1, BUB1B, CASC5, CCNB1, CCNB2, CCNE2, CEP55, CKAP2L, DLGAP5, DTL, E2F8, ECT2, ESCO2, EXO1, EXOC6, FBXO5, FIGNL1, HELLS, HMMR, IARS, KIF11, KIF18A, KIF20A, KNTC1, MAD2L1, MASTL, MTHFD2, NCAPG2, NCAPH, NEIL3, NUF2, PRC1, PSAT1, RTKN2	0,64	0,23	1,67	0,370
Activated CD8	ADRM1, AHSA1, C1GALT1C1, CCT5, CCT6B, CETN3, CSE1L, EIF2S1, GAL, GEMIN6, GPT2, KIAA0101, MND1, MPZL1, MRPS16, PCNA, PTRH2, RFC5, SPC25, TIMM13, TIMM8B, TK1, TUBB, TXNDC17	1,2	0,46	3,53	0,710
Central memory CD4	ADAM19, AHNAK, AIM2, COL4A1, CYLD, DPEP2, EPHA4, FYN, IL6R, IPCEF1, KLF10, MAL, MALT1, NXN, OPTN, PLCL2, PNRC1, SMAD4, SPOCK2, TRIB2, TXK	0,96	0,35	2,51	0,930
Central memory CD8	ADAM23, ATP2B4, BCL2L11, CD300A, CD80, CHN1, F2R, F8, GABARAPL1, GPR183, KLRK1, MSC, PYHIN1, RCAN2, RGS1, SLAMF1, SLFN11, ZEB2	1,6	0,6	5,39	0,380
Cytotoxic cells	BST2, BTN3A2, IL10RA, KLRB1, LAT2, LILRA2, SELL, SLAMF6, SLAMF7	5,06	1,48	31,66	0,030
DC	C1QC, CCDC88A, CCL13, CCL3L1, CCL3L3, CD300E, CD86, CLEC1A, CLEC4C, CLEC5A, CLIC2, CSF2RA, FAM49A, FCGR1A, FN1, FSTL1, GPR109B, GPX3, HLA-DQA2, INHBA, LGMN, NGFR, PDGFRL, PDPN, PRKAR2B, SIGLEC1, SIGLEC5, SLAMF9, STAB1, THBD, TNFAIP2, TTYH2, UBD, VCAM1	1,2	0,44	3,16	0,700
Effector memory CD4	BMI1, CASP3, CNOT10, COPB2, DARS, EXOSC9, EZH2, GDE1, IFT74, KLF5, NDUFB9, NUP205, SHCBP1, XRCC6	0,55	0,17	1,46	0,250
Effector memory CD8	APOBEC3H, ARHGAP10, ATP10D, BIRC3, C3AR1, CCL4, CD244, CD55, CFLAR, CMKLR1, DAPP1, DFNB31, DRAM1, EFNA5, FCGR2C, FCGR3A, FCRL6, FGR, GPR114, GZMH, HAPLN3, HLA-DMB, HLA-DPA1, HLA-DPB1, IFI16, IKZF2, IL15, IL2RB, JAKMIP1, KLRD1, LY96, NFKBIA, PLXNC1, PPP4R1, PTPN22, RGS18, SETD7, SH2D1B, SLA2, STXBP1, TLR5, TYROBP	1,94	0,75	5,86	0,190
Eosinophil	CCL24, CFD, CREB3, EGR1, EPX, FBXO16, HFE, HIVEP1, HIVEP2, MBP, PRNP, RNASE2, RNASE3, S100A4, SIGLEC8	1,14	0,42	2,97	0,780
iDC	ATP6V1A, C1QB, CHST11, CSF1, DPYD, FNDC3B, GRP, IL1RN, INPP5F, LPL, LPXN, MMD, PLAU, PLCB2, RAB38, RDX, RRAGD, TACSTD2, TGM2, TIMD4, TM7SF4	1,39	0,53	4,19	0,520
Immature B cells	BANK1, CD22, CYBB, ETS1, FAM129C, FCRL1, FCRL2, FCRL3, FCRL5, FCRLA, HDAC9, HLA-	1,87	0,7	6,24	0,240

Supplementary Table S4. Logistic regression analysis results of immune cell phenotypes enrichment scores as predictors of treatment response.

Immune Cell population	Gene	Odds Ratio	conf.low	conf.high	p.value
	DOB, HLA-DQA1, HVCN1, KIAA0226, NCF1, NCF1B, P2RY10, PNOC, SP100, STAP1, TAGAP, TXNIP, ZCCHC2				
Macrophages	AIF1, CCL1, CCL14, CCL26, CD163, CD300LB, CNR1, CNR2, CPM, CSF3R, ENG, FCAR, IGF1, IL34, L1CAM, LILRA1, LILRA5, LRP1, MS4A7, MS4A8B, TREM1, TREML1	1,43	0,55	3,91	0,460
Mast cells	ACSL4, ADAMTS3, ADCYAP1, ARHGAP15, ATP8B4, C19orf59, CASQ1, CLC, CMA1, CPA3, CTSG, DUSP14, EGR3, EMR1, FST, HDC, HEY1, KRT1, LXN, MEIS2, MS4A2, MS4A3, NLRP3, NTRK1, PLAT, PTGS1, PTGS2, RGS16, SCG2, SDPR, SERPINB2, SIGLEC14, SIGLEC6, SLC18A2, SLC24A3, TAL1, TARP, TIE1, TIMP1, TNFAIP6, TSPAN4, VAT1	0,96	0,35	2,56	0,930
mDC	ATP5O, CBX1, DNAJC15, ENSA, FOXN3, PRCP, SSB, TPMT	0,74	0,26	1,95	0,550
MDSC	ADORA3, AG2, ARG1, BIN2, C1orf162, CAPS, CD117, CD11B, CD11C, CD124, CD14, CD15, CD163L1, CD1D1, CD21, CD23, CD274, CD31, CD35, CD40, CD43, CD44, CD66B, COX2, CR2, CTR9, EBP, FAM48A, FAM70B, FCER2, FCGRT, FERMT3, FLOT1, FLT1, GIMAP7, GLI4, GNA15, GPR34, GPSM3, HLA-DR, IDO, IKZF1, IL12, IL13, IL18BP, IL1R, IL4RA, INPP5D, ITGA3, KDR, KRIT1, LGALS3, MGAT4A, NAIP, NEK3, NFSF13, NOG, PARVG, PDRG1, PECAM1, PIK3R5, PPP1R2P4, PSAP, PTGES2, PTPRE, RNASE1, RP11, S100A8, S100A9, SELPLG, SLA, SLC36A1, SLC44A1, ST8SIA4, STAT3, STAT6, TBXAS1, TFGFB1, TFGFB2, TFGFB3, TFGFB5, TFRC, TGFB2, TPP1, VTCN1	2,14	0,76	7,88	0,190
Memory B cells	AICDA, CCNA2, CDKN3, CLCN5, ENPP1, FCER1A, FCRL4, MYC, RUNX2, SORL1, SOX5, STAT5A, STAT5B, TLR9	1,12	0,43	3,26	0,820
Monocytes	ACTR3, ANXA5, ARPC2, ATP6V1B2, BASP1, CD300LF, DAZAP2, EIF4A1, EIF4G2, EMP3, FCN1, FTL, GABARAP, HIF1A, LITAF, NCOA4, OLR1, RAB1A, RHOA, SAT1, SDCBP, SRGN, TEK, TMBIM6, UBE2D3	1,3	0,49	3,54	0,580
Neutrophils	ABTB1, AMPD2, CAMP, EMR4P, FPR1, FPR2, GPR77, MAEA, PROK2, SEC14L1, SEPX1, SLC25A37, TNFSF14, TREML4, VNN2, XPO6	1,15	0,44	3,24	0,780
NK	AKT3, AXL, CDH2, CRTAM, CYTH1, FASLG, GRB2, KLRG1, LILRB5, LST1, MAPK4, NOTCH3, PIK3CG, PILRA, PLCG2, SIGLEC7, SIGLEC9	1,58	0,61	4,66	0,350
NK56 bright	C11orf75, DYNLL1, HCP5, HDGFRP2, KRT86, MLST8, MYL6B, TAX1BP3	1,92	0,7	7,14	0,250
NK56 dim	AKR7A3, CLTB, FAM27A, GLS2, GPRC5C, GRIN1, HLA-E, KIR2DS4, KLHL21, KRT80, MPL, PHRF1, PORCN, PQBP1, PSMC4, TEX264, UPP1	1,95	0,71	7,04	0,230
NKT	BTN2A2, CD101, GNLY, KIR2DL1, KIR2DL3, KIR3DL1, KIR3DL2, KLRC1, MICB, NCR1, NFATC2IP, TNFRSF11A	2,41	0,9	8,76	0,110

Supplementary Table S4. Logistic regression analysis results of immune cell phenotypes enrichment scores as predictors of treatment response.

Immune Cell population	Gene	Odds Ratio	conf.low	conf.high	p.value
pDC	CBX6, DAB2, DDX17, HIGD1A, IDH3A, IL3RA, MAGED1, NUCB2, OFD1, OGT, PDIA4, SERTAD2, SIRPA, SPCS3, TMED2, TMX1, UGCG, ZDHHC17	0,74	0,28	1,95	0,530
T cells	BATF, BTLA, CCL11, CCL17, CCL22, CCL3, CCL5, CCL7, CCR1, CCR3, CCR4, CCR8, CD1A, CD1B, CD1C, CD1D, CD1E, CD247, CD28, CD3D, CD3E, CD3G, CD69, CD8A, CXCR1, CXCR4, CXCR5, CXCR6, FGFBP2, FLT3, FOSL1, GRAP2, HLA-DRA, HLA-DRB1, HLA-DRB5, HRH2, IFNG, IL10, IL12B, IL12RB1, IL12RB2, IL18R1, IL18RAP, IL1B, IL21, IL21R, IL24, IL26, IL27, IL2RA, IL6, IL7, IRF4, IRF8, ITGAL, ITK, JAK2, JAK3, LAIR1, LAT, LAX1, LCK, LCP2, LILRA3, LILRA4, LILRB1, LILRB2, LILRB3, LILRB4, MS4A4A, NAPSB, NFATC1, NFKB2, P2RY14, PIK3CD, POU2F2, PRF1, RUNX3, SPI1, STAT1, STAT4, TNF, TPRG1, TSLP, UBASH3B, ZAP70	2,66	0,91	11,16	0,110
TFH	B3GAT1, BCL6, CCR7, CD200, CD83, CD84, CDK5R1, FGF2, GPR18, PDCD1	1,44	0,55	3,99	0,450
TGD	ACP5, C1orf54, CARD8, CCL18, CD209, CD33, CECR1, CLEC10A, CLEC4A, CSF1R, FCER1G, FCGR2A, FCGR2B, FCGR3B, FGF7, FGL2, FPR3, GM2A, GPNMB, HCK, HK3, LIPA, MARCO, MMP12, MNDA, MRC1, MS4A6A, NPL, PLA2G7, PLEK, RAB23, RNASE6, SDC2, SLAMF8, SLC15A3, SLC38A6, SLC7A7, SPP1, TFEC, TM6SF1, TNFSF8, TREM2, TRPV2	1,49	0,57	4,15	0,410
Th1	ADAM8, CCR5, CD151, CD48, CD52, CD53, CD6, CD68, CD7, CD96, CD97, EBI3, GPR84, HAVCR2, ICAM1, ICAM3, IGSF6, IL12A, IL7R, IRF1, ITGA4, ITGAM, ITGAX, ITGB2, ITGB4, ITGB7, LTA, LTB, METRNL, P2RX5, PTPRC, SPN, TBX21, TLR6, TNFRSF1A, TNFRSF9, TRAT1	3,1	0,96	15,68	0,100
Th17	ABP1, B4GALNT4, C2CD4A, C2CD4B, CA2, CCDC65, CEACAM3, CHRM3, DOC2B, F12, FURIN, GPR25, IL17A, IL17C, IL17F, IL17RC, IL17RE, IL23A, ILDR1, LONRF3, LTK, MOCOS, SH2D6, TNIP2, TRAF3IP2, YBX2	1,1	0,42	3,19	0,850
Th2	ASB2, CALD1, CCR2, CSRP2, DAPK1, DLC1, DNAJC12, DUSP6, GATA3, GNAI1, HTR2B, LAMP3, NRP2, OSBPL1A, PDE4B, PHLDA1, PLA2G4A, RAB27B, RBMS3, RNF125, SIGLEC10, SKAP1, SMAD2, TMPRSS3, UBASH3A	0,87	0,31	2,29	0,780
Treg	CCL19, CD34, CD72, CTLA4, FOXP3, GADD45B, GEM, IL1RL1, IL9R, MADCAM1, MYH10, NCF2, RCSD1, RYR1, SELE, SELP, SFRP1, SIT1, TIGIT, TLR10, TLR2, TLR7, TLR8, TRAF1, WIPF1, TGFB1	1,83	0,7	5,54	0,230
